# Synthetic-polymer-assisted antisense oligonucleotide delivery: targeted approaches for precision disease treatment

**DOI:** 10.3762/bjnano.16.34

**Published:** 2025-03-27

**Authors:** Ana Cubillo Alvarez, Dylan Maguire, Ruairí P Brannigan

**Affiliations:** 1 School of Chemical Sciences, Dublin City University, Glasnevin, Dublin 9, Ireland

**Keywords:** antisense oligonucleotides, enhanced delivery, gene transfection, intracellular uptake, locked nucleic acid (LNA), nanoparticles, peptide nucleic acid (PNA), personalised therapy, phosphorodiamidate morpholino oligomer (PMO), phosphorothioate (PS), polyplexes, ribose substitutions, small interfering RNA (siRNA), synthetic polymers, tricyclo-DNA (tcDNA)

## Abstract

This review explores the recent advancements in polymer-assisted delivery systems for antisense oligonucleotides (ASOs) and their potential in precision disease treatment. Synthetic polymers have shown significant promise in enhancing the delivery, stability, and therapeutic efficacy of ASOs by addressing key challenges such as cellular uptake, endosomal escape, and reducing cytotoxicity. The review highlights key studies from the past decade demonstrating how these polymers improve gene silencing efficiencies, particularly in cancer and neurodegenerative disease models. Despite the progress achieved, barriers such as immunogenicity, delivery limitations, and scalability still need to be overcome for broader clinical application. Emerging strategies, including stimuli-responsive polymers and advanced nanoparticle systems, offer potential solutions to these challenges. The review underscores the transformative potential of polymer-enhanced ASO delivery in personalised medicine, emphasising the importance of continued innovation to optimise ASO-based therapeutics for more precise and effective disease treatments.

## Introduction

The development and use of personalised therapies tailored to individual patients have emerged as a powerful strategy for treating various oncological, autoimmune, and infectious conditions, where genetic expression plays a crucial role on the disease pathogenesis and prognosis [1–4]. In the past decades, several approaches including cell-based therapies [5–7], gene therapies [8–9], and RNA therapies [10–11] have been studied to target diseases and genetic variations. However, the lack of disease-specific biomarkers, which facilitate the identification of disease phenotypes, the poor chemical stability of nucleotide-based molecules and their inefficient biodistribution and targeted delivery result in insufficient biological activity and immune-related adverse effects, limiting their clinical application [12–13]. Antisense oligonucleotide-based therapies have garnered great attention as precision disease treatments because of their increased target specificity and resistance to nuclease degradation, as well as enhanced cellular internalisation [14].

**Figure 1:**
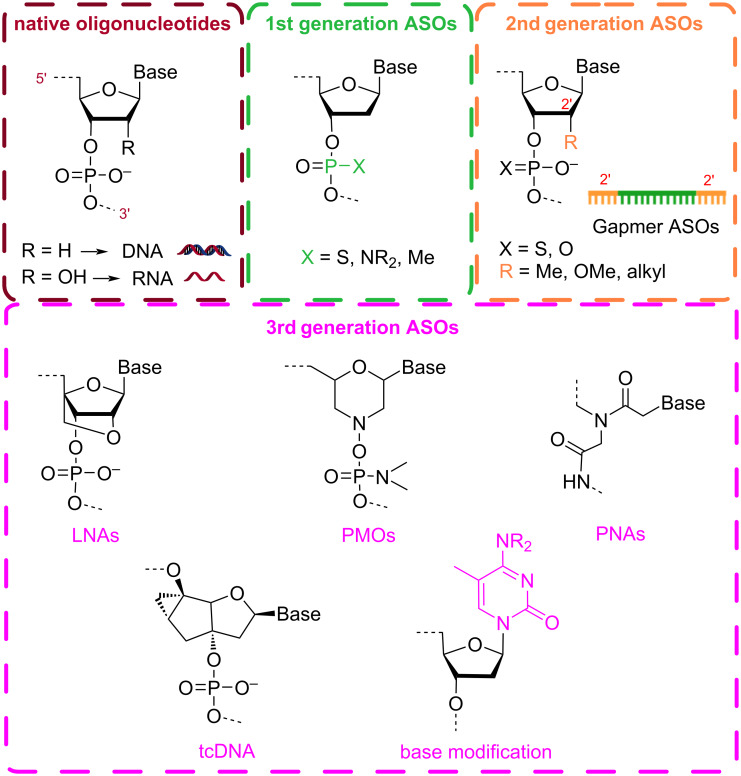
Common chemical modifications used in antisense oligonucleotides (ASOs). First-generation ASOs involve modifications in the natural phosphodiester linkage found in native oligonucleotides (ODNs). Second-generation ASOs introduce changes at the 2′-*O*-position of the ribose sugar and are commonly designed as gapmer ASOs. Third-generation ASOs encompass more advance chemistries, including locked nucleic acids (LNAs), phosphorodiamidate morpholino oligomers (PMOs), peptide nucleic acids (PNAs), tricyclo-DNA oligomers (tcDNA), and nucleotides with synthetic bases.

Antisense oligonucleotides (ASOs) are short, chemically engineered single- or double-stranded oligonucleotides (also known as small interfering RNA, siRNA) that bind to complementary RNA in a sequence-specific manner, causing the suppression, modification, or restoration of a specific protein [15]. Since their discovery in 1978 by Zamecnik and Stephenson [16], design and synthesis of novel ASOs have been developed extensively with the aim to improve their biostability, pharmacokinetics, and intracellular accumulation. To date these synthetic oligonucleotides can be classified into three generations based on their structural modifications (Figure 1) [17]. First-generation ASOs are characterised by having an altered phosphate backbone. This chemical modification increases their stability against nucleases, extending their half-life, and promotes the degradation of transcripts by RNase H. Nevertheless, the low binding affinities to their target RNA can trigger immunostimulatory response and cause off-target effects [18]. To overcome these limitations, newer generations of antisense nucleotides were designed to improve binding affinity and hybridisation stability with target messenger RNA (mRNA). This reduces immune-related adverse events and toxicity while keeping increased resistance to nuclease degradation. In second-generation ASOs, the structural modifications are not limited to the phosphate linkage; the molecules also possess alkyl substitutions at the 2′ position of the ribose ring, whereas third-generation ASOs are more diverse, containing a variety of sugar ring modifications or base modifications [19–20]. These include locked nucleic acids (LNAs) [21–22], phosphorodiamidate morpholino oligomers (PMOs) [23–25], peptide nucleic acids (PNAs) [26–27], and tricyclo-DNA oligomers (tcDNA) [28–29]. It is well-established that 2′-modifications inhibit the ribonuclease RNase H1 to cleave the target mRNA, which restricts the use of 2′-*O*-substituted oligoribonucleotides as antisense agents [30]. In order to overcome this limitation, chimeric gapmer oligonucleotides, in which a short DNA sequence or a first-generation ASO is capped on both ends with second-generation ASOs, have been developed and combine the increase in binding affinity of 2′-modified residues with the RNase H activation from the DNA central region [31–32].

**Figure 2:**
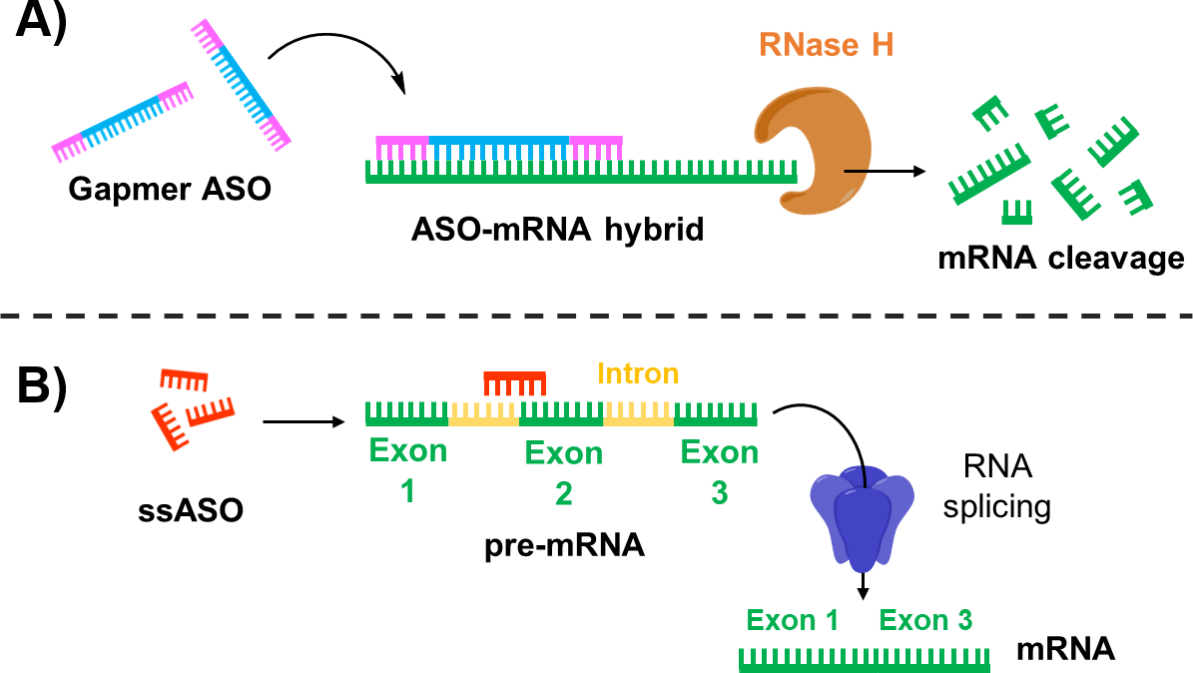
Mechanisms of action of ASOs. (A) RNase H-recruiting ASOs through formation of ASO–mRNA hybrids that can be recognised by RNase H. (B) Splice switching ASOs (ssASOs) modulate splicing processes by targeting pre-mRNA.

According to their chemistry and design, ASOs can regulate protein expression by either knocking down mRNA transcripts or modulating the pre-mRNA splicing process (Figure 2). RNase H-dependent ASOs promote mRNA cleavage by forming stable RNA–DNA hybrids, which serve as enzymatic substrate for RNase H activation, thereby reducing RNA transcription and protein translation (Figure 2A) [33]. This category includes certain phosphorothioate ASO drugs such as fomivirsen, which inhibits the replication of human cytomegalovirus, as well as several 2′-methoxyethyl-modified gapmers such as mipomersen, inotersen, and volanesorsen, which are used to treat of homozygous familial hypercholesterolemia, hereditary transthyretin-mediated amyloidosis, and familial chylomicronemia syndrome, respectively [34]. Additionally, some modified ODNs have been designed to possess intrinsic enzymatic activity through the incorporation of ribozymes and DNAzymes, which directly catalyse the cleavage of targeted mRNA, resulting in transcript knockdown [27,35]. Alternatively, ASOs can be engineered to bind specific intron–exon junctions of a targeted pre-mRNA, influencing the exclusion or inclusion of splicing enhancers and silencers (Figure 2B). By doing so, it can restore or disrupt reading frames as well as alter protein transcription levels [36]. For example, splice switching ASOs (ssASOs) have been proven to efficiently mask polyadenylation signals [37] and hamper intron excision on pre-mRNA [38]. This prevents the formation of mature mRNA, causing a downregulation of protein synthesis. Two recently approved antisense drugs that use this approach include eteplirsen and nusinersen. The former is a phosphorodiamidate morpholino-derived oligomer designed to treat Duchenne muscular dystrophy (DMD) by skipping exon 51 during dystrophin mRNA processing [39]. The latter ssASO drug contains 2′-*O*-2-methoxyethyl modifications, which allow for the inclusion of exon 7 in the gene SMN2. This promotes the production of SMN protein, ameliorating the symptoms of spinal muscular atrophy [40].

ASO-based therapeutics have demonstrated great potential in the expansion of patient-personalised therapies [41–42]. In 2019, Kim et al. pioneered the development of milasen, a ssASO drug tailored to a single patient affected by a rare form of Batten’s disease, and its clinical efficiency was successfully proven [43]. Despite significant progress in the discovery of novel oligonucleotide drugs, their poor biodistribution and intracellular delivery have limited their use as therapeutic agents. In fact, efficient delivery to most tissues other than liver, kidneys, lungs, retina, brain, and spinal cord poses major challenges because of the broad systemic distribution, the extensive amounts of tissue that need to be targeted to achieve a therapeutic response, and the large degree of tissue-to-tissue variability [44–47].

Negatively charged ASOs including phosphorothioate backbone oligonucleotides (PS), gapmer ASOs, tcDNA, and LNAs can interact with plasma proteins, which reduces their rate of renal clearance and influences their distribution to target tissues [48]. In contrast, neutral ASOs such as PMOs and PNAs present lower binding affinity to plasma proteins; therefore, these molecules exhibit shorter circulation lifetimes and lower tissue uptake [49]. After making contact with the targeted cells, ASO drugs still need to be internalised by cells and overcome internal cellular trafficking to determine their functional delivery. During this process the oligonucleotides are engulfed into endosomes and released into the cytoplasm, where they either exert their action or continue to the cellular nucleus. Certain cell surface receptors have demonstrated efficient binding to modified oligonucleotides, including integrins [50], toll-like receptors [51], and scavenger receptors [52]. However, the intracellular delivery to their specific site of action remains the major barrier in ASO-based therapies. Some promising strategies to overcome these limitations involve the conjugation of ASOs with peptides [53], polymers [54], aptamers [55], and antibodies [56], as well as the development of novel drug delivery systems [57–58].

This review discusses the challenges associated with delivering antisense drugs and explores multiple synthetic-polymer-based strategies developed to enhance target tissue specificity and minimise off-target effects. In particular, we focus on synthetic polymer-based methodologies to deliver ASO therapeutics across biological barriers and their potential use in precision medicine over the past decade.

## Review

### Synthetic polymers for enhanced ASOs delivery

Polymer-assisted platforms for enhanced delivery have attracted considerable attention because of their versatile drug delivery capabilities and their diverse chemical composition, which enables post-polymerisation modification for drug conjugation or additional targeting [59]. In addition to small molecules, drugs, and proteins, polymers play an essential role in the delivery of nucleic acids as they provide high stability and flexibility [60]. The delivery or nucleic acids can be improved either by direct conjugation to carriers or through their incorporation into nanoparticle-based complexes. Various synthetic polymers used for targeted ASO delivery include poly(amino acids), polyamines, polyacrylates and polyolefins, and neutral polymers.

#### Poly(amino acids)

**Figure 3:**
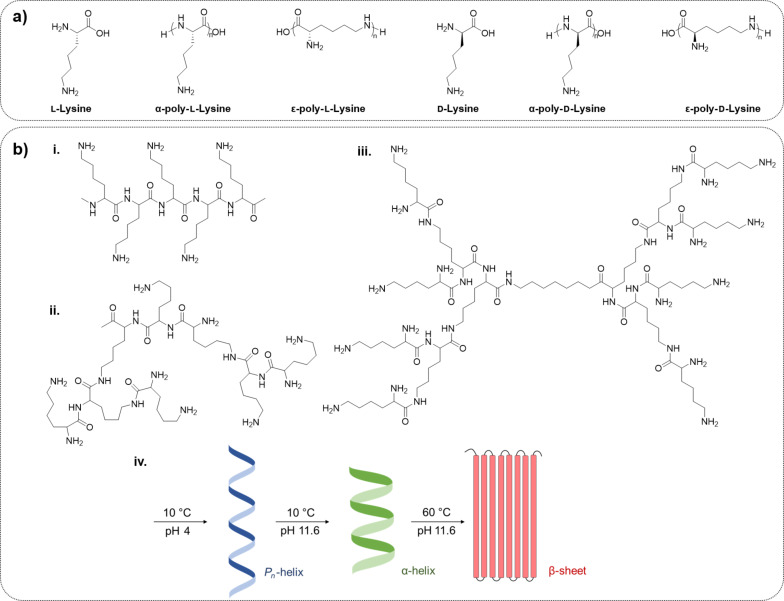
Schematic representation of poly(ʟ-lysine) (PLL). (a) Chemical structure of lysine monomers. (b) (i) Linear, (ii) dendritic, and (iii) hyper-branched architectures of PLL, and (iv) configuration of PLL.

**Poly(ʟ-lysine).** Poly(ʟ-lysine) (PLL) is a cationic biopolymer, commonly employed in the delivery of ASOs because of its ability to facilitate enhanced cellular uptake [61]. Depending on desired molecular weight (*M*_w_), molecular architecture, and molar-mass polymer dispersity (*Ð*_M_), PLL is traditionally synthesised through three synthetic approaches, that is, chemical–enzymatic synthesis (CES), solid-phase peptide synthesis (SPPS), and ring-opening polymerisation (ROP) of *N*-carboxy anhydrides [62]. While CES and SPPS offer access to structural isomers (i.e., α-PLL or ε-PLL) and sequence-controlled ʟ-lysine-rich peptides, respectively, NCA-ROP is the favoured synthetic method for the synthesis of high-molecular-weight PLL with a wide range of polymer architectures such as linear, dendritic, and highly branched, while maintaining low polydispersity (Figure 3) [63–64].

In their 2020 study, Shen et al. utilised ε-poly(ʟ-lysine) (εPLL) (≈4.2 kDa) as a cationic polymer to enhance the delivery of single-strand phosphorothioate backbone ASOs (ASO-GAPDH, ASO-PHD2, and ASO-Luci) through the formation of green nanoparticles (GNPs) with natural polyphenols such as epigallocatechin gallate [65]. The authors demonstrated that εPLL played a crucial role in the successful coating of these nanoparticles, significantly increasing their zeta potential and improving cellular uptake. Moreover, εPLL-coated GNPs were found to provide robust protection for the ASOs against nuclease degradation, maintaining over 78% of the oligonucleotides intact after RNase treatment. In addition, the εPLL-modified GNPs exhibited high gene-silencing activity in HeLa-Luc cells, indicating efficient delivery of ASOs and other splice switching oligonucleotides, such as anti-microRNAs and DNAzymes, into the cells. The following year, Le Vay et al. designed a PLL-based system to enhance ribozyme-catalysed RNA recombination and oligonucleotide assembly within peptide–RNA condensates [27]. This work demonstrated that low-molecular-weight PLL (*M*_w_ = 1–5 kDa) facilitated charge-mediated phase separation, significantly shifting the reaction equilibrium from RNA cleavage to ligation. This shift promoted the isothermal assembly of long and complex RNA molecules from short fragments under mild conditions, offering a potential model for early catalytic processes in prebiotic environments. The study highlighted the ability of PLL to support and enhance ribozyme activity and nucleic acid catalysis, effectively bridging the gap between short oligomers and functional RNAs under various environmental conditions.

In addition to a delivery enhancer, PLL can be efficiently employed as an antisense oligonucleotide condenser. In their exploration of advanced drug delivery systems for cancer therapy, Kim et al. employed PLL in the synthesis of dually stabilised triblock copolymer micelles for the systemic delivery of phosphorothioate ASOs (metastasis associated lung adenocarcinoma transcript 1 lncRNA-targeted ASO (*MALAT1*-ASO) and GL3 luciferase-targeted ASO (*GL3*-ASO)) targeting solid tumours [66]. Triblock copolymers comprising poly(2-ethyl-2-oxazoline) (PEtOx), poly(2-*n*-propyl-2-oxazoline) (PnPrOx), and PLL (*M*_w_ = 6.9 kDa, degree of polymerization (DP) = 42) segments allowed for the formation of compartmentalised micelles bearing a hydrophilic PEtOx shell, a thermoresponsive PnPrOx interlayer, and an ASO/PLL polyion complex (PIC) core. These cationic micelleplexes exhibited enhanced stability in serum-containing media and prolonged blood circulation, leading to a more efficient accumulation of ASO payloads in a prostate cancer xenograft model. Notably, this approach significantly improved gene silencing efficiency in prostate cancer cells, compared to a control diblock copolymer micelle.

**Figure 4:**
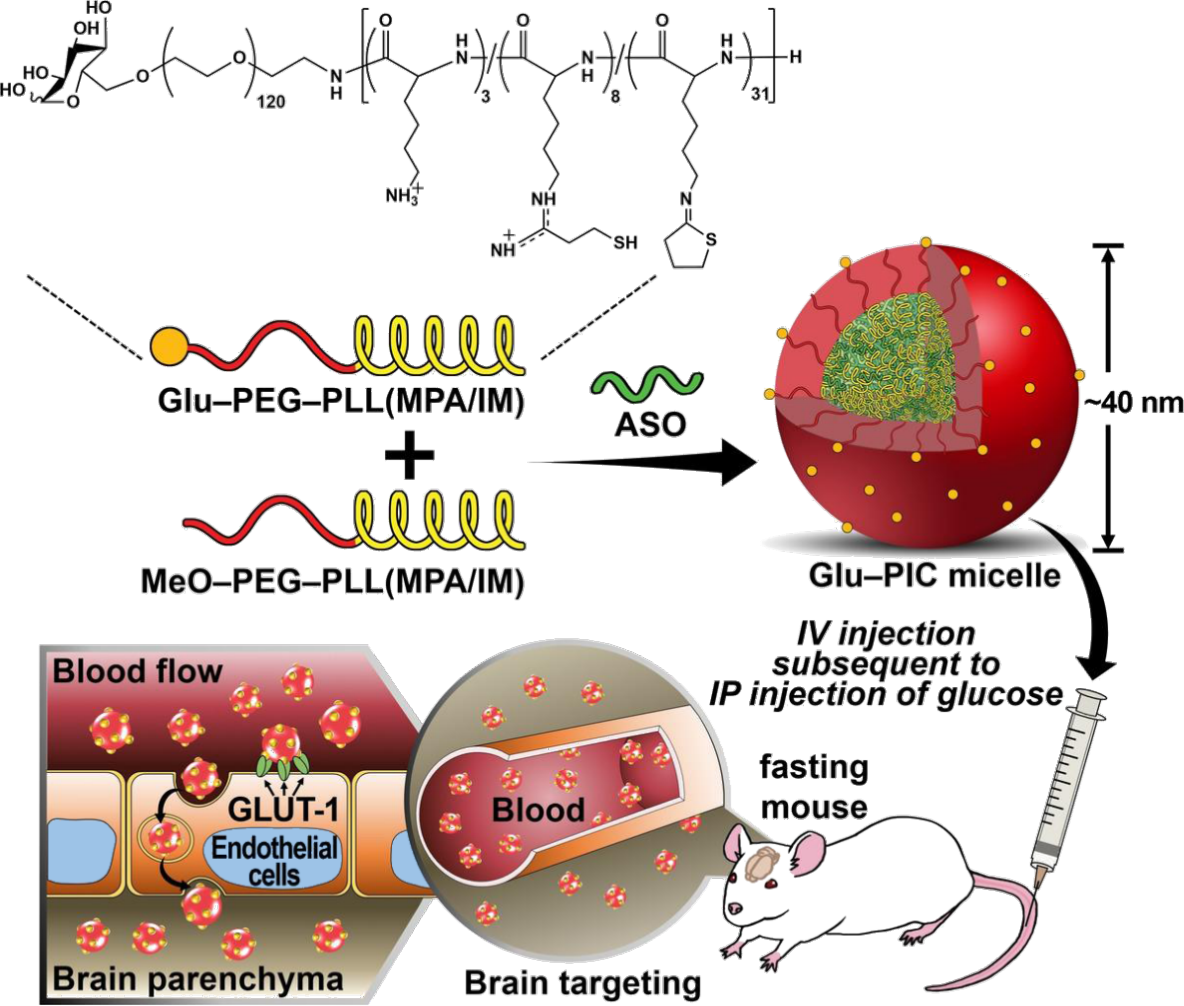
Illustration of the delivery of ASO-loaded glucosylated-polyion complex micelles (Glu-PIC/Ms) to the brain. This figure was reproduced from [71] (© 2020 Hyun Su Min et al. Published by Wiley-VCH Verlag GmbH & Co. KGaA, distributed under the terms of the Creative Commons Attribution-Noncommercial 4.0 International License, https://creativecommons.org/licenses/by-nc/4.0/). This content is not subject to CC BY 4.0.

While cellular internalisation of ASO complexes can take place through several pathways, it has been previously reported that non-specific adsorptive endocytosis is the predominant internalisation mechanism for PLL-conjugated ASOs [67]. Specific molecular structures, however, can be utilised to facilitate the targeted uptake of drug delivery systems. Ligand–PLL–ASO conjugates, for instance, can enter target cells via receptor-mediated endocytosis [68]. This approach holds promise for precision disease treatment, as certain receptors for growth factors, hormones, and vitamins are often overexpressed in defective cells. The incorporation of moieties such as proteins, sugars, folic acid, steroids, and growth factors provide an effective means of targeting and enhancing cellular uptake of conjugated drugs or drug delivery systems. For example, early studies by Zheng et al. demonstrated a strategic use of PLL conjugated to galactose (Gal–PLL, Gal/PLL = 10:1) as a carrier for a 16-mer phosphorothioate analogue of the antisense oligodeoxynucleotide 5′-CATGCCCCAAAGCCAC-3′, targeting the hepatitis B virus (HBV) [69]. The conjugation significantly enhanced the delivery and efficacy of the ASO in inhibiting HBV gene expression. The study showed that the Gal–PLL–ASO complex effectively reduced HBsAg and HBeAg levels in HBV-transfected cells by 96% and 82%, respectively, compared to 70% and 58% inhibition by the ASO alone. Furthermore, the Gal–PLL conjugation facilitated higher liver-specific uptake, with a 52.1% concentration in the liver compared to 21.9% for ASOs alone. This targeted approach resulted in a significant reduction in HBV DNA levels in transgenic mice, with some achieving undetectable levels after treatment. Employing a similarly galactosylated PLL system, Jing et al. explored a novel approach using Gal–PLL (*M*_w_ = 48 kDa, Gal/PLL = 24:1) to enhance the targeted delivery of *c-myc* antisense oligodeoxynucleotides to hepatocellular carcinoma (HCC) cells via ultrasound-targeted microbubble destruction [70]. In this instance, Gal–PLL was used as a targeting ligand because of its ability to bind to asialoglycoprotein receptors on liver cells, promoting specific uptake by the HCC cells. The study’s results showed that the combination of Gal–PLL–ASO with SonoVue microbubbles significantly suppressed *c-myc* mRNA expression, reduced cell proliferation, and inhibited tumour growth both in vitro and in vivo, compared to other groups without Gal–PLL or SonoVue. This demonstrated the essential role of Gal–PLL in enhancing the specificity and efficiency of antisense delivery. In a separate study, Min et al. investigated the use of PLL to develop glucose-coated polymeric nanocarriers for the systemic delivery of ASOs across the blood–brain barrier (BBB) [71]. The authors utilised a polyion complex micelle (PIC/M) platform based on poly(ethylene glycol)-*b*-poly(ʟ-lysine) (PEG–PLL, DP_PLL_ = 42) modified with 3-mercaptopropyl amidine and 2-thiolaneimine (Figure 4). This design allowed for stable encapsulation of a LNA-modified gapmer structure with a phosphorothioate linkage backbone targeting the *MALAT1* gene. The presence of disulfide cross-links in the micelle core was found to enhance particle stability in the bloodstream and enabled the controlled release of *MALAT1* ASO in the brain’s reductive environment. As a result, significant knockdown of targeted long non-coding RNA was observed in key brain regions, including the cerebral cortex and hippocampus, after a single intravenous administration. These results underscored the potential of these nanocarriers as a non-invasive method for effective ASO delivery to the brain, offering a promising strategy for treating central nervous system disorders.

Besides glycosylation, the utilisation of targeting sequenced peptides has also gained attention. Ou et al. investigated the use of a modified PLL as a carrier for the targeted delivery of the radioiodinated 15-mer phosphorothioate ASO 5′-AACGTTGAGGGGCAT-3′ (^131^I-ASO) to HT29 human colon cancer xenografts [72]. In this study, PLL (*M*_w_ = 3 kDa) was conjugated to vasoactive intestinal peptide (VIP) in a 1:1 molar ratio to enhance the delivery of ^131^I-ASO to VIP-receptor-positive tumour cells. The authors found that conjugating the radioiodinated ASO to the VIP-PLL significantly improved tumour uptake and reduced plasma clearance, resulting in a 5.6-fold decrease in plasma clearance and a 3.4-fold increase in tumour uptake compared to unconjugated ^131^I-ASO. Moreover, the treatment with VIP-PLL ^131^I-ASO conjugate demonstrated strong antitumor effects, decreasing tumour growth rates up to 9.67-fold more effectively than unconjugated ^131^I-ASO. It is worth mentioning that while the ASO-conjugate exhibited moderate toxicity, particularly in terms of decreased white blood cell and platelet counts, these negative effects remained within acceptable ranges, suggesting that VIP–PLL conjugation could effectively enhance the therapeutic efficacy of the ASO while maintaining manageable toxicity levels.

In addition to chemical functionality, studies have demonstrated the importance of the architectural structure of the PLL employed [73–74]. In a study on the delivery of the ASO 5′-TGGCGTCTTCCATTT-3′ (PS-ODN) to two cell lines, D407 (clone 6-2) and CV-1 (clone CV-11), both expressing luciferase mRNA and activity, Jääskeläinen et al. demonstrated that linear PLLs with average molecular weights (*M*_n_) of 4, 20, and 200 kDa exhibited no enhancement on PS-ODN activity [75]. Conversely, Marano et al. showed that a series of hybrid lipid–lysine dendrimer–ASO conjugates resulted in 40–60% reduction in VEGF expression over 24 h, when applied to the same human RPE cell line, D407 [76]. These results demonstrated that dendrimers with higher densities of positive charges (specifically high-generation dendrimers containing eight charges) significantly enhanced transfection efficiency, achieving up to 81.5% compared to 56.8% for dendrimers with fewer charges. This improved transfection efficiency correlated with a significant reduction in VEGF expression, with certain dendrimer/ODN-1 complexes maintaining VEGF inhibition for up to 48 h after transfection. Moreover, in vivo experiments using a rat model of laser-induced choroidal neovascularization (CNV) showed that specific dendrimer/ODN-1 complexes, particularly those formed with dendrimers 4 and 7, significantly inhibited CNV development, reducing the severity of vascular leakage for up to two months. Similarly, Eom et al. presented an investigation into the application of dendritic poly(ʟ-lysine) (DPL) polymers as carriers for ASO delivery, highlighting their great potential due to their cationic nature and structural versatility [77]. The study systematically evaluated various generations of DPLs, demonstrating that higher-generation DPLs (e.g., generations 5 and 6) significantly improved the delivery efficiency of ASOs into HeLa cells, as evidenced by increased luciferase activity in a splicing correction assay. This was attributed to the higher charge density and molecular size of the higher-generation DPLs, which facilitated more effective complexation with ASOs and enhanced cellular uptake. Additionally, while these high-generation DPLs exhibited moderate cytotoxicity, complexation with ASOs was shown to reduce toxicity, making them a promising vehicle for gene therapy applications. Confocal microscopy further confirmed the ability of these DPL–ASO complexes to achieve nuclear localisation, although some challenges remain regarding endosomal escape and complete nuclear delivery. Le et al. recently investigated the targeted delivery of ASOs through the conjugation of cyclic RGD and iRGD peptides to the DPL structure [78]. This modification was designed to enhance integrin receptor-mediated uptake in target cells, and the study demonstrated that DPL–RGD and DPL–iRGD conjugates effectively formed complexes with ASOs, facilitating their cellular delivery. While the conjugates initially showed moderate cellular toxicity, this was significantly reduced following their complexation with ASOs. Enhanced delivery efficiency was further confirmed using a splicing correction assay, where the optimal dose of the DPL–ASO complex resulted in substantial luciferase activity, indicating successful nuclear delivery of the ASOs.

Even though endocytosis enables cellular entry, achieving efficient release and nuclear delivery of ASOs remains a challenge as a consequence of potential sequestration and degradation within endocytic vesicles [79–80]. This issue is prevalent in the majority of therapeutic delivery systems that enter via endocytosis and limits the therapeutic efficacy of ASOs as nuclear access is often crucial for their intended function. Therefore, strategies focused on promoting endosomal escape and enhancing nuclear delivery are crucial for improving the uptake efficiency of lysine-conjugated ASOs and maximising their therapeutic potential [81]. Utilising cyclohexene-1,2-dicarboxylic anhydride (DCA)-modified PLL dendrigrafts (DGL), Shen et al. focused on enhancing the lysosomal escape of ASOs [82]. The authors demonstrated that DCA modification significantly improved the lysosomal escape capability of the nanocomposite, and the successful delivery of ASOs into HepG2 and HeLa cells, with the modified DGL–DCA nanocomposites showing superior performance in promoting lysosomal escape compared to unmodified versions. Additionally, the analysis of HIF-1α mRNA expression levels and HIF-1α protein assays confirmed that the nanocomposite effectively inhibited both mRNA expression and protein translation synchronously; it also exhibited a significant tumour inhibitory rate of 77.99% in vivo, underscoring the potential of this delivery system for antitumor therapy.

**Poly(ʟ-ornithine).** Although similar in structure, there has been a relative scarcity of research into poly(ʟ-ornithine) (PLO) as a transfection agent when compared to poly(ʟ-lysine) [83–84]. While PLL has a well-established track record of effectiveness in transfection, PLO has traditionally been used more for enhancing cell attachment and growth in culture rather than for transfection, limiting its exploration in gene delivery [85]. Furthermore, while both PLO and PLL can induce cytotoxicity at elevated concentration, PLL’s toxicity has been more thoroughly studied and mitigated. This success has made PLL the preferred choice in many studies, reducing the impetus to explore alternatives such as PLO [86]. It should be noted that, ornithine-rich sequenced peptides and oligoornithine (4–8 repeat units) have successfully been employed in the delivery of ASOs [87–88]; however, this is outside of the scope of this review.

Fimmel et al. examined the use of PLO as a transfection agent for introducing ASOs into human keratinocytes [89]. The authors determined that PLO was particularly effective in facilitating the uptake of ASOs, with the optimal concentration being 12 µg/mL in a serum-free medium. The transfection process, followed by a brief dimethyl sulfoxide shock, allowed for efficient delivery of ASOs into the cytoplasm of keratinocytes without significantly affecting the expression of key genes, such as androgen receptor and β-actin. This study highlighted poly(ʟ-ornithine) as a promising tool for enhancing ASO delivery in epithelial skin cells while maintaining cell viability, demonstrating its potential for therapeutic applications in dermatology. More recently, Taniguchi et al. explored the use of PLO as a key component in a novel drug delivery system designed for the treatment of advanced breast and pancreatic cancers [90]. The researchers developed a branched PEGylated poly-ʟ-ornithine (PEG-PLO) to form a unit polyion complex (uPIC) with PRDM14-specific chimeric siRNA, which was administered intravenously to mouse models. PEG-PLO exhibited longer retention times in the bloodstream and enhanced accumulation in tumour tissues, leading to a significant reduction in tumour size and metastasis in both orthotopic and xenograft models. Importantly, the PEG-PLO-based delivery system showed a lower nitrogen-to-phosphate ratio requirement compared to similar systems using PEG-PLL, thereby reducing the amount of polymer needed for effective siRNA delivery. The results indicated that PEG-PLO not only facilitated effective tumour suppression but also prolonged survival in treated mice without causing severe toxicity.

**Figure 5:**
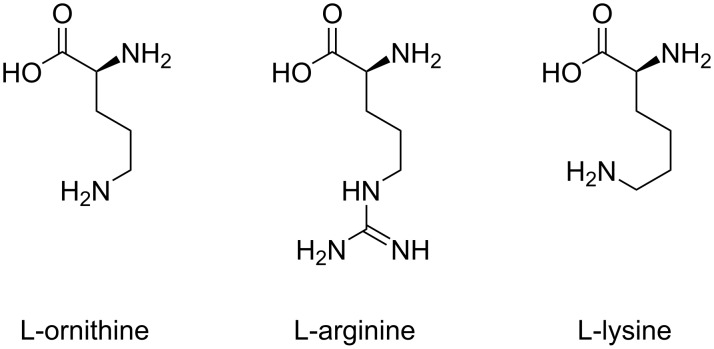
Structure of the cationic amino acids, ʟ-ornithine, ʟ-arginine, and ʟ-lysine.

**Poly(ʟ-arginine).** Poly(ʟ-arginine) (PLR) is a cationic polypeptide composed of repeating units of the amino acid ʟ-arginine (Figure 5). Thanks to its characteristic amphipathic guanidinium group, PLR has been frequently used in biomedical applications, particularly as a carrier for nucleic acid delivery [91]. Moreover, its strong cationic nature allows it to effectively bind to negatively charged molecules, facilitating cellular uptake through endocytosis. Additionally, PLR has been studied for its ability to enhance cell penetration and promote endosomal escape, which are crucial steps for the intracellular delivery of therapeutic agents [92–93]. However, like other cationic poly(amino acids), its use is sometimes limited by potential cytotoxicity, requiring careful optimisation for therapeutic applications.

**Figure 6:**
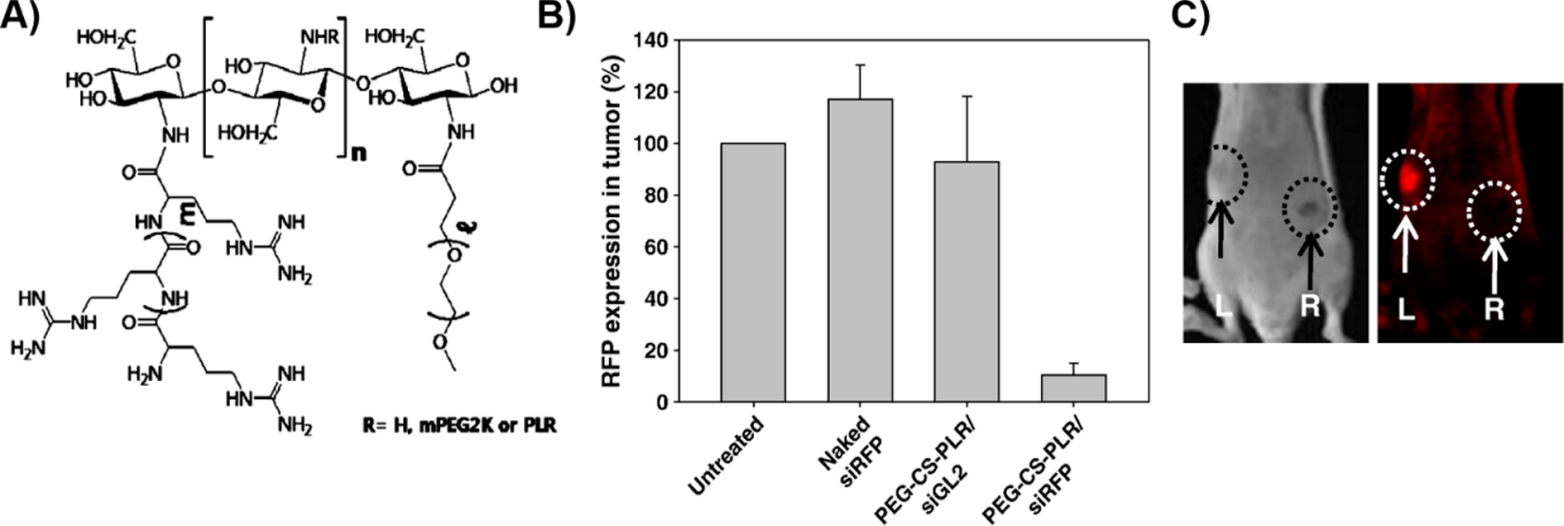
(A) Chemical structure of PEGylated poly(ʟ-arginine)–chitosan derivatives (PEG-CS-PLR) polymers. (B) Expression of targeted RFP protein in siRNA-treated tumours and untreated tumours. (C) Molecular imaging of tumour-bearing mice after siRNA treatment with PEG-CS-PLR. Figure 6 was adapted from [95], *Journal of Controlled Release*, Volume 145, Issue 2, Noh, S. M.; Park, M. O.; Shim, G.; Han, S. E.; Lee, H. Y.; Huh, J. H.; Kim, M. S.; Choi, J. J.; Kim, K.; Kwon, I. C.; Kim, J.-S.; Baek, K.-H.; Oh, Y.-K., “Pegylated poly-ʟ-arginine derivatives of chitosan for effective delivery of siRNA”, Pages 159–164, Copyright (2010), with permission from Elsevier. This content is not subject to CC BY 4.0.

In their pursuit of more effective gene delivery systems, Kim et al. explored the potential of poly(ʟ-arginine) as a vital component in targeting CD44-positive cancer cells through a novel siRNA delivery platform [94]. The researchers complexed PLR (*M*_w_ = 15–70 kDa) with hyaluronic acid (HA) to form biodegradable nanoparticles capable of delivering siRNA to cells overexpressing the HA receptor CD44. The study demonstrated that these HA–PLR complexes, in particular the formulation HP101, were able to form stable complexes with siRNA and exhibited enhanced cellular uptake in CD44-positive cells, such as WM266.4 melanoma cells. As a result, HP101 significantly reduced the expression of target genes at both mRNA and protein levels, indicating successful RNA interference. Additionally, the administration of HP101 complexes demonstrated lower cytotoxicity compared to poly(ʟ-arginine) alone, with a 23-fold higher TC_50_ value observed for HP101. In a similar study, Noh et al. investigated the use of PLR as a key component in a novel siRNA delivery system [95]. PLR was grafted onto chitosan (CS) and further PEGylated to enhance delivery efficiency and reduce cytotoxicity. The study revealed that the PEGylated PLR–CS conjugate (PEG-CS-PLR) formed stable complexes with siRNA and significantly improved cellular uptake compared to CS and PLR alone. Notably, PEG-CS-PLR demonstrated enhanced serum stability and reduced hemolysis, which are critical for in vivo applications. When administered intratumorally, PEG-CS-PLR–siRNA complexes efficiently silenced target gene expression in tumour tissues, outperforming other delivery systems, including non-PEGylated variants (Figure 6). In an effort to improve targeted gene therapy for head and neck cancer, utilising a similar strategy to the 2009 Kim et al. study, Cho et al. developed a PLR and dextran sulfate (DEX)-based nanocomplex for the delivery of epidermal growth factor receptor (EGFR) siRNA [96]. The study demonstrated that this PLR–DEX nanocomplex, which was optimised at a 15:1 weight ratio, significantly enhanced cellular uptake of EGFR siRNA in Hep-2 and FaDu cells, leading to efficient gene silencing, with up to 95.2% reduction in EGFR expression observed in Hep-2 cells. Additionally, in vivo experiments using a FaDu tumour xenograft mouse model showed that the PLR-DEX/siRNA complex effectively inhibited tumour growth, outperforming both naked siRNA and PLR–siRNA complexes.

In 2012, Cheng and Saltzman coated biodegradable polymer nanoparticles with nona-arginine (ARG), an oligomeric form of poly(ʟ-arginine), to enhance the cellular uptake and delivery efficiency of PMOs [97]. The study demonstrated that ARG-coated poly(lactide-*co*-glycolide) (PLGA) nanoparticles significantly improved the internalisation of ASOs into cancer cells, leading to effective inhibition of miR-155, a known oncogene, and alteration of the splicing pattern of the Mcl-1 gene, promoting the pro-apoptotic isoform. In following studies, Zhao et al. developed a novel siRNA delivery system using PLR as a condensing block in the formation of cationic micellar nanoparticles [98]. In this report, biodegradable amphiphilic triblock copolymers composed of monomethoxy poly(ethylene glycol) (mPEG), poly(ᴅ,ʟ-lactide) (PLA), and PLR (DP = 15) were self-assembled into micellar nanoparticles that could effectively bind anti-EGFR siRNA (antisense strand: 5′-AUGUUGCUUCUCUUAAUUCCUdTdT-3′), forming stable “micelleplexes”. The study demonstrated that these PLR-based micelleplexes exhibited significant gene silencing efficiency, with approximately 65% inhibition of EGFR expression in MCF-7 cells. This was comparable to the performance of Lipofectamine 2000, a commercial transfection reagent. Furthermore, the micelleplexes showed minimal cytotoxicity and hemolytic activity, making them a promising and safer alternative for in vivo siRNA delivery in tumour therapy.

**Poly(ʟ-glutamic acid).** Poly(ʟ-glutamic acid) (PLG) is a biodegradable, water-soluble synthetic polymer composed of repeating units of the amino acid ʟ-glutamic acid. Owing to the presence of the carboxyl side chains, PLG is anionic under physiological conditions (pH ≈ 7.4). This property allows PLG to be used primarily as a stabilising carrier or a controlled-release matrix for drug and ASO delivery [99]. Even though its negative charge often limits direct complexation with negatively charged ASOs, it can be used in conjugation strategies or in combination with other cationic materials to increase colloidal stability and enhance retention of ASOs in targeted tissues [100]. Despite its versatility and potential, research focused on the use of PLG as an antisense delivery system is relatively limited compared to its cationic counterparts.

In 2008, Sun et al. investigated the use of PLG (DP = 20) in the development of a novel gene delivery system. In this study, surface-modified complexes were designed using PLG as a backbone to which polyethylene glycol (PEG, *M*_w_ = 5, 10, and 20 kDa) or epidermal growth factor (EGF) were conjugated [101]. This approach aimed to enhance the stability and transfection efficiency of DNA–PLL complexes. The study found that the incorporation of PLG significantly improved the performance of the gene delivery system, with the PLG-conjugated complexes achieving up to 25-fold higher transfection efficiency compared to unmodified complexes. Although this study focused on the utilisation of oligonucleotides, these findings highlight the potential of PLG as a versatile and effective component in non-viral gene delivery platforms, particularly in maintaining stability and enhancing efficiency in environments containing serum. Subsequently, Ma et al. employed PLG (*M*_w_ ≈ 56 kDa) as a synthetic polymer to conjugate with an amino-terminated CpG oligonucleotide (5′-EEGGGACGATCGTCEEEEG-3′-NH_2_) for targeted cancer immunotherapy, and demonstrated its effectiveness in a mouse model of melanoma [102]. These results showed that PG-CpG significantly enhanced the retention of CpG in both tumours and draining lymph nodes following intratumoral injection. Specifically, the study found that 48 h after injection, a notable percentage of PG-CpG remained in the tumour (26.5%) and in the draining inguinal lymph nodes (1.5%), compared to much lower retention rates with free CpG. This prolonged retention translated into potent antitumor efficacy, as PG-CpG triggered robust activation of antigen-specific CD8+ T-cell responses and significantly inhibited tumour growth.

#### Polyamines

**Figure 7:**
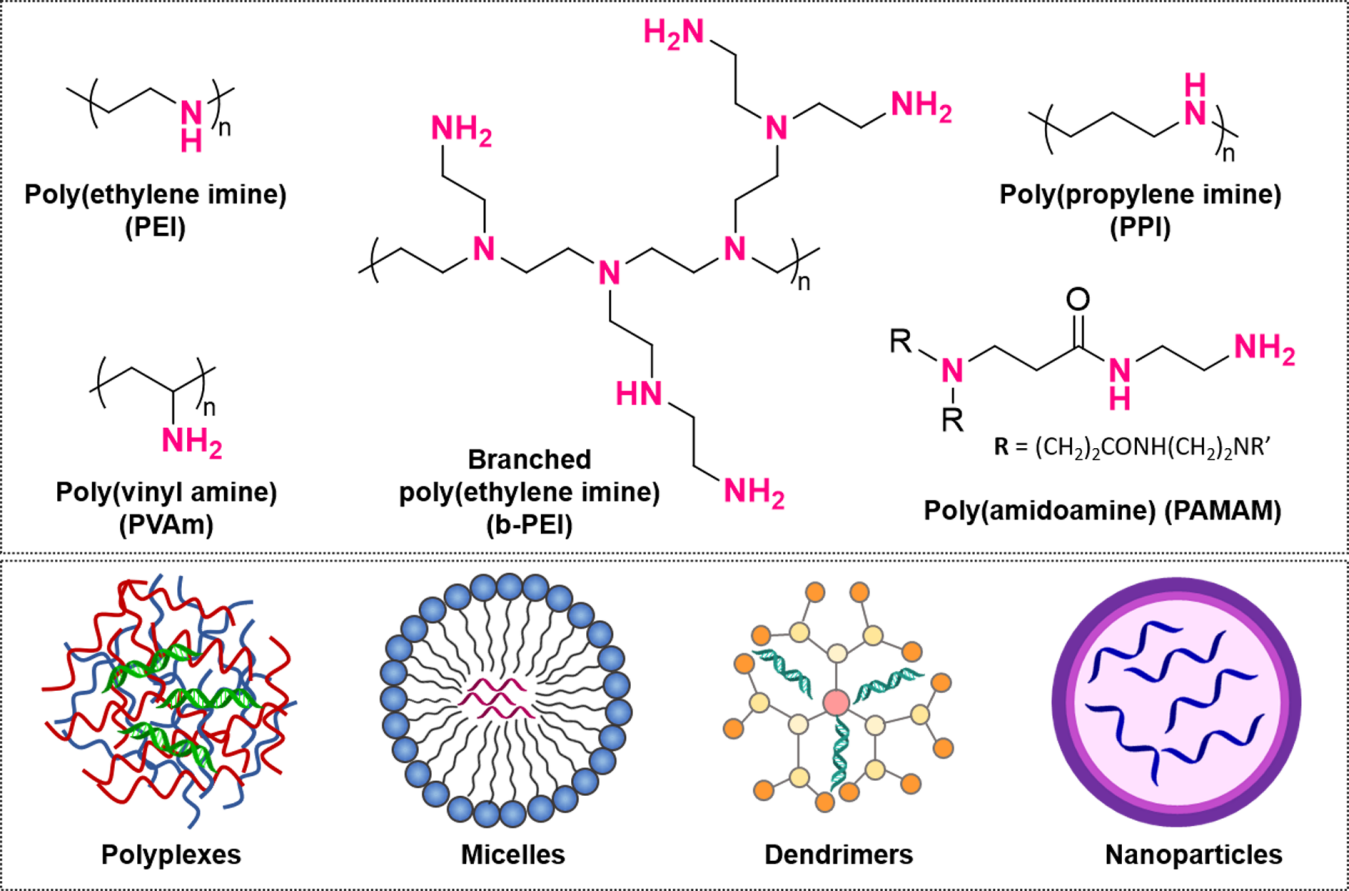
Polyamines frequently employed in ASO delivery systems include linear and branched poly(ethylene imine) (PEI and b-PEI respectively), poly(propylene imine) (PPI), poly(amidoamine) (PAMAM), and poly(vinyl amine) (PVAm); along with a representation of different particle types often used to deliver antisense and siRNA oligonucleotides.

Polyamines are among the most extensively studied polymeric materials for assisted ASO delivery because of their ability to overcome biological barriers (Figure 7) [103]. These polymers are characterised for containing multiple amine groups (–NH_2_) along their backbone, which readily become positively charged in aqueous environments. This positive charge enables strong electrostatic binding with negatively charged nucleic acids and enhances cellular uptake, leading to increased accumulation of therapeutic agents at the target site [104]. Extensive literature regarding the biodistribution and cell internalisation of polyamine-based carriers has shown that factors such as the molecular weight [105–106], architecture [107–108], degree of amine/phosphate complexation [109], and surface charge density of the polymers [110] strongly affect the transfection efficiency and toxicity of these cationic platforms. In this section of the review, we discuss the progress and challenges associated with the use of multiple polyamines for antisense delivery.

**Poly(ethylene imine).** Previous studies have shown that the incorporation of cationic poly(ethylene imine) (PEI) in lipid nanocarrier formulations enhanced the cellular internalisation of ASOs and siRNA, resulting in increased transfection activity [111]. Thanks to the ability of PEI to efficiently bind negatively charged macromolecules and overcome cellular barriers, PEI-based systems have been extensively investigated for targeted antisense oligonucleotide and gene delivery. In 2011, Yang et al. studied the bioactivity in vitro and in vivo of lipid–PEI_2000_–DNA (sLPD) nanoparticles conjugated with a phosphorothioate ASO (G3139), designed for Bcl-2 downregulation in cancer cells [112]. The reaction between PEI and hexadecenal on the lipidic bilayer enabled the formation of a reversible Schiff’s base, which provided higher particle stability. sLPD assemblies exhibited higher colloidal stability and cellular internalisation compared to free G3139 and non-stabilised counterparts. As a result, significantly higher inhibition of Bcl-2 proteins was observed in sLPD-treated groups, which led to induced apoptosis of KB cells and increased antitumour activity in xenograft mouse models. Later experiments investigated the effect resulting from the incorporation of targeting agents into the surface of lipid–polycation vectors. For example, Yuan et al. prepared transferrin-conjugated PEI_1200_–lipid nanoparticles (LPNs), for the targeted delivery of G3139 to acute myeloid leukemic cells [113]. Agarose gel electrophoresis retardation essays revealed that both PEI and transferrin were critical to achieve nanocomplexes with long-term stability and optimal cargo protection under physiological conditions. Moreover, LPNs exhibited enhanced cellular uptake and more effective Bcl-2 downregulation than naked G3139 and non-PEI-containing nanoparticles, leading to prolonged tumour growth suppression. In a similar study, Zheng et al. explored the effect of high-molecular-weight PEI (*M*_w_ = 25 kDa) on the biostability, cytotoxicity, and transfection activity of a series of transferrin-conjugated LPNs with varied physicochemical properties, designed to deliver a phosphorothioate ASO targeting ribonucleotide nuclease R1 [114]. Experimental and in vitro studies indicated that optimal biostability and cytocompatibility were achieved with particles bearing a nitrogen-to-phosphate (N/P) ratio of 6:1 and moderate zeta potentials (ζ = 15 mV), while maintaining high suppression rates of the R1 protein. Alternatively, Sleiman and coworkers studied the cytotoxicity and gene silencing activity of anti-luciferase phosphorothioate ASOs (Luc-ASO) complexed to DNA–polymer conjugates with various compositions and architectures [115]. PEI–DNA complexes with various N/P ratios were prepared using linear PEI of different molecular weights (2.5, 5.0, and 25.0 kDa) and b-PEI (*M*_w_ = 25.0 kDa), followed by their conjugation with either free Luc-ASO, dodecane-ASO (HE_12_-Luc-ASO), or (dodecane-*co-*hexaethylene glycol)-ASO ((HE-HEG)_6_-Luc-ASO). Linear PEI complexes demonstrated high biocompatibility even at high molecular weights, whereas b-PEI induced significant cytotoxicity. Moreover, the cell-uptake efficiency was improved by increasing the N/P ratio. The bioactivity of the conjugates was found strongly influenced by the molecular weight of the polycation. Complexes formed with PEI_2.5kDa_ and PEI_5kDa_ exhibited lower transfection efficiency and poor gene silencing compared to PEI_25kDa_ complexes. In addition, the introduction of dodecane diol polymers played a crucial role in gene silencing since it enabled the formation of (Luc-ASO-HE_12_):PEI–DNA micelles, which provided greater stability and protection of antisense nucleotides. This resulted in enhanced cell uptake and transfection activity when compared to free ASO and Luc-ASO-(HE-HEG)_6_ complexes.

Additionally, the attachment of hydrophobic moieties, amino acids, and small peptides to linear and branched PEI has been explored as an alternative to improve the biostability and bioavailability of cationic polyplexes while offering a targeted delivery with reduced cytotoxicity that is frequently associated with high-molecular-weight PEI (*M*_w_ ≈ 25 kDa) [116]. Early studies by Kim et al. [117] and Xie et al. [118] investigated the targeted modulatory effects of siRNAs and ASOs delivered using branched and linear PEI carriers conjugated with folate and linoleic acid, respectively. In both reports, the conjugated carriers demonstrated greater transfection activity and protein silencing while keeping low levels of cytotoxicity compared to naked and oligonucleotide–PEI complexes. This improvement was attributed to the unsaturated nature of the hydrophobic moieties in the conjugates. In another study, Cheng and coworkers synthesised a library of bPEI polyplexes grafted with hydrophobic ligands including aliphatic alkanes, fluoroalkanes, and cycloalkanes of varying lengths and analysed the structure–activity relationship of these compounds to deliver anti-luciferase siRNA oligonucleotides [119]. All PEI-modified vectors showed enhanced gene knockdown in vitro compared to unmodified PEI, regardless of the hydrophobic ligand. Notably, bioactivity was found to be directly correlated to alkane chain length, and fluoroalkylated systems generally outperformed their alkene and cycloalkene counterparts, achieving luciferase inhibition efficacies exceeding 80%. The modification of high-molecular-weight PEI with amino acids such as leucine, tryptophan, tyrosine, and phenylalanine has also proven advantageous in delivering small RNA oligonucleotides [120]. In particular, tyrosine-conjugated PEI_25kDa_–ssASO polyplexes exhibited greater splice correction effectiveness on mRNA and protein levels, measured by the increase in luciferase activity on engineered HeLa cells containing an altered luciferase gene. In later reports, Yang et al. demonstrated that bPEI–lipid nanoparticles functionalised with small cell-penetrating peptides on their surface significantly improved the intracellular delivery of LOR-2501, a negatively charged phosphorothioate ASO [121].

Another strategy to improve colloidal stability, decrease cytotoxicity and immune response, and reduce the renal clearance of PEI delivery systems is through the incorporation of hydrophilic moieties such as PEG –see section “Neutral polymers”–and poly(ethylene oxide)-*b*-poly(propylene oxide)-*b*-poly(ethylene oxide) (PEO-*b*-PPO-*b*-PEO) triblock copolymers (Pluronic^®^), which are known to induce structural rearrangements in cellular membranes [122]. Belenkov et al. examined the antitumoral activity of Ku86 ASOs complexed with Pluronic (P85)–PEI (2 kDa) conjugates [123]. In vitro studies revealed a significant reduction in Ku86 protein levels and increased apoptosis rates following radiotherapy in cells treated with these cationic polyplexes. Moreover, experimental studies in mice with colon adenocarcinoma xenografts showed a marked reduction in tumour growth after the co-administration of Ku86–P85–PEI polyplexes and ionising radiation, which demonstrated the synergistic therapeutic effect of the system. Similarly, Vinogradov et al. reported the differences in biostability and bioactivity of various ASO-conjugated Pluronic–PEI (2 kDa) complexes with different hydrophilic–lipophilic balances (HLBs), focusing on three Pluronic polymers, namely, P38, P85, and P123 (ranging from hydrophilic to highly hydrophobic) [124]. All mixed copolymer systems exhibited similar cytotoxicity, with around 50% cell survival at a concentration of 100 mg/mL. Polyplexes containing the most hydrophilic Pluronic (P38) exhibited the highest ASO loading efficiency but suffered from limited cell uptake because of their high polarity. In contrast, P123–PEI micelles showed superior internalisation rates but were ineffective inhibiting MDRI-mRNA because of their inability to escape internal endosomes. The ASO-conjugated P85–PEI polyplexes displayed an optimal balance between transcytosis and endocytosis, resulting in efficient ASO internalisation and subsequent mRNA regulation.

**Figure 8:**
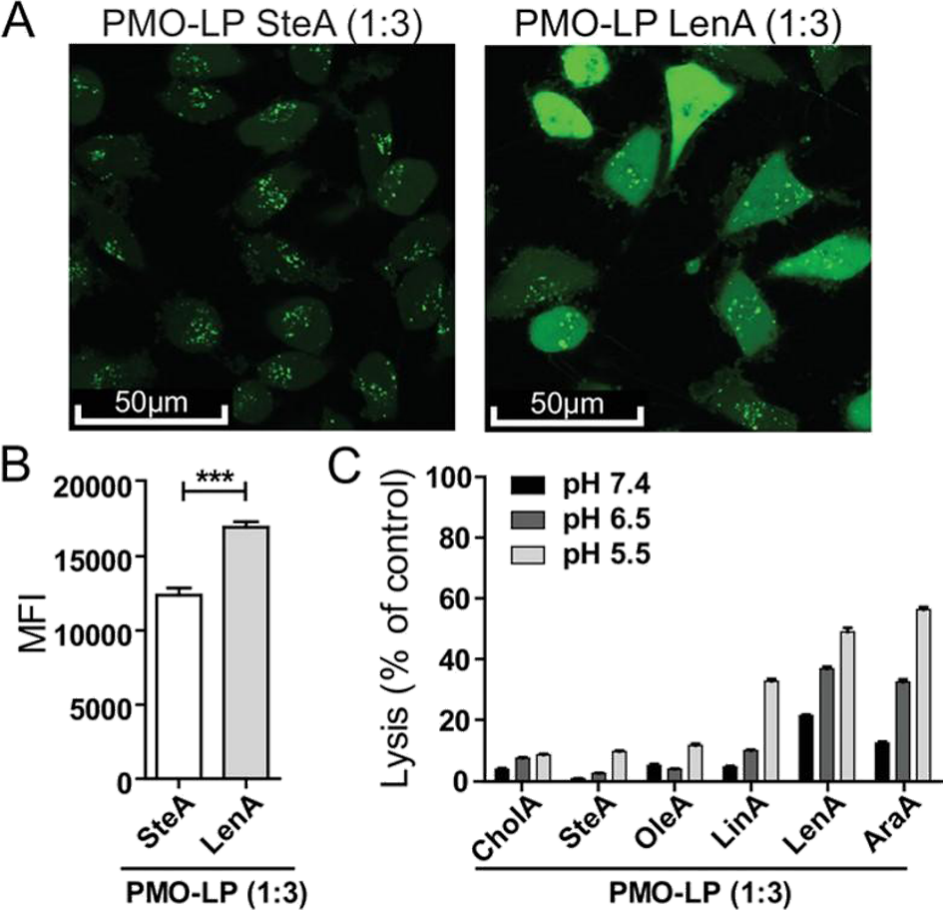
Impact of unsaturated fatty acids on cellular membrane interactions. (A) Confocal microscopy images of HeLa pLuc/705 cells treated with 0.45 mg·mL^−1^ calcein and PMO-LP SteA (control) or PMO-LP LenA at a concentration of 5 × 10^−6^ M for 4 h. (B) Cellular calcein fluorescence intensity determined by flow cytometry. (C) Haemoglobin levels after 60 min of incubation with PMO conjugates (2.5 × 10^−6^ M) at pH 7.4, 6.5, and 5.5. Figure 8 was reproduced from [127] (© 2019 Jasmin Kuhn et al. Published by WILEY-VCH Verlag GmbH & Co. KGaA, Weinheim, distributed under the terms of the Creative Commons Attribution 4.0 International License, https://creativecommons.org/licenses/by/4.0/).

All aforementioned studies demonstrated the great potential of cationic PEI–ASO vehicles for antisense therapy since the positively charged surface facilitates antisense conjugation and interaction with cellular membranes. However, the conjugation and in vivo delivery and uptake efficacy of neutral ASOs, such as PMOs and PNAs, have proven challenging and significantly less effective [125]. To overcome this limitation, research has focused on the design of novel platforms where the therapeutic oligonucleotides are covalently attached to the cationic polymer through reversible linkages, rather than simply forming complexes. Berthold et al. prepared PNA-functionalised PEI systems (branched, 25 kDa) by reacting the terminal amine groups in PEI with *N*-succinimidyl-3-(2-pyridyldithio)propionate (SPDP) and SPDP–dPEG_8_–NHS (NHS = *N*-hydroxysuccinimide), which provide chemical handles for the reversible attachment of cysteine-PNAs via disulfide linkage [126]. These compounds showed a PNA dose-dependent cell toxicity and exhibited up to almost 20-fold increased luciferase activity compared to free arginine-PNAs and dithiothreitol control. In another study, Kuhn et al. developed aminoethylene-based PMO conjugates through strain-promoted azide–alkyne cycloaddition of dibenzocyclooctyne-modified PMOs and azide-containing artificial peptides, previously conjugated to small cationic oligo(ethylenamino) blocks containing fatty acid units as hydrophobic moiety for polyplex stabilisation [127]. In this study, the PMO conjugate containing linolenic acid with three double bonds (LenA) exhibited enhanced cellular internalisation while maintaining high cell viability on various cell lines, leading to the highest splice switching activity (Figure 8).

**Figure 9:**
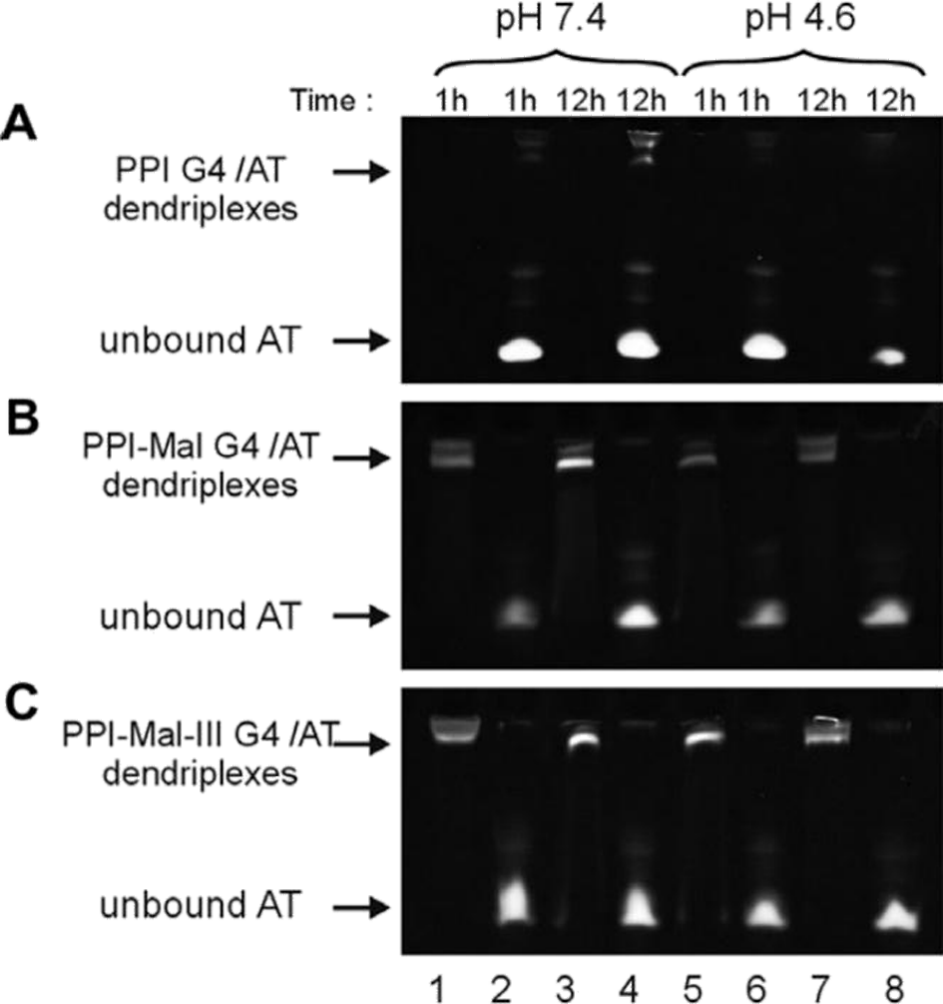
Stability of dendriplexes composed of GEM91 and (A) PPI G4, (B) PPI-Mal G4, and (C) PPI-Mal-III G4. This figure was reprinted from [135], *Biochemical and Biophysical Research Communications*, Volume 427, Issue 1, J. Drzewińska; D. Appelhans; B. Voit; M. Bryszewska; B. Klajnert, “Poly(propylene imine) dendrimers modified with maltose or maltotriose protect phosphorothioate oligodeoxynucleotides against nuclease activity”, Pages 197–201, Copyright (2012), with permission from Elsevier. This content is not subject to CC BY 4.0.

**Poly(propylene imine).** Since pioneering research by Vögtle and coworkers [128], study and synthesis of poly(propylene imine) (PPI) dendrimers for drug and gene delivery have grown exponentially [129–130]. PPI dendrimers are highly branched, tree-like macromolecules with well-defined structures and dense positively surface charge comprised of a backbone of tertiary tris(propylene) amines that facilitate nucleotide escape from the endosomes through a “proton sponge” effect, whereas primary amines are found in the periphery, which enable further particle functionalisation and effective complexation with nucleic acids [131]. Hollins et al. analysed the efficacy of low-generation PPI dendrimers (G2 and G3) for the delivery of ODNs targeting the EGFR to human epidermoid carcinoma cells [132]. Both dendrimer formulations demonstrated enhanced cellular uptake into both cytoplasm and nuclei, as well as efficient anti-EGFR activity (up to 60%) compared to free ASO and oligofectamine–ASO complexes; cytotoxicity appeared to be related to dendrimer generation and, therefore, their molecular weight. Later studies by Ziemba et al. confirmed that the genotoxicity of PPI dendrimers was surface charge-dependent through the analysis of fourth-generation dendrimers (PPI G4) with low and high surface charge density, that is, open shell and dense shell, respectively [133]. In addition, reduced systemic cytotoxicity was achieved through partial modification of PPI G4 with oligosaccharides such as maltose (Mal) and maltotriose (Mal-III), while maintaining optimal apoptosis rates in human cancer cell lines [134]. Klajnert and coworkers further explored the ability of Mal and Mal-III-modified PPI G4 dendrimers to protect anti-HIV ASOs from nucleolytic degradation under physiological conditions (Figure 9) [135]. Similarly, Maly et al. investigated the self-assembly process of PPI-Mal G4 and PPI-Mal-III G4 dendrimers conjugated with multiple anti-HIV ASOs. Dendriplexes with diverse sizes and morphologies, including 1D rod-like and 3D diamond-shaped structures, were successfully prepared by altering the physicochemical properties of the carriers and the oligonucleotide type [136].

In addition, PPI dendrimers have also proven advantageous in delivering antisense siRNAs to targeted tissues [137–138]. Taratula et al. reported the development of siRNA nanoparticles from reversible-cross-linked PPI dendrimers (G5) bearing disulfide linkages, which enable a controlled release through disulfide bond cleavage [139]. Following dendrimer PEGylation (PEG5000 modified with a synthetic luteinizing-hormone-releasing hormone (LHRH) analogue) provided greater particle stability and active targeting to specific cancer cells, which led to increased intratumoral accumulation and enhanced suppression of Bcl-2 mRNA. In recent studies, Mal-modified PPI G4 dendrimers conjugated to single chain fragment variables were designed to exclusively deliver anti-EGFR siRNA to tumours by receptor-mediated endocytosis [140].

**Poly(amidoamine).** In the past decades, poly(amidoamine) (PAMAM) dendrimers have gained significant attention as a versatile platform for antisense delivery, largely owing to their superior biocompatibility, biodegradability, and ASO-loading capabilities compared to other cationic systems including PEI and PPI dendrimers [141]. Moreover, the presence of primary amine groups in the surface of PAMAM dendrimers enable their post-assembly functionalisation with cell-penetrating peptides or molecules of therapeutic interest such as fatty acids, which enhance the delivery capabilities of ASO-modified dendrimers [142–143]. The cytotoxicity of these systems can further be reduced through PEGylation, which offers significant advantages by addressing common limitations of dendrimer-based systems such as drug leakage, immunogenicity, and poor ASO solubility [144]. Patil et al. demonstrated that PAMAM-*b*-PEG_3000_-*b*-PLL–siRNA triblock copolymers exhibited reduced cytotoxicity and improved intracellular delivery of Bcl-2 siRNAs compared to PLL–siRNA and PEG-*b*-PLL–siRNA nanocarriers [145]. Nowadays, because of the straightforward synthesis and commercial availability, PAMAM dendrimers are considered the main dendritic system for nucleic acid delivery and gene transfer [146].

Extensive research has focused on the development of novel antisense PAMAM dendriplexes to fight cancer processes. Various reports from Nourazarian and Najar demonstrated that the co-administration of PAMAM dendrimers loaded with anti-EGFR and c-Src oligonucleotides effectively downregulated both protein and gene expression, thereby inhibiting the proliferation of targeted colon cancer cells [147–149]. In another study, Venuganti et al. prepared hydrogels containing anti-Bcl-2-PAMAM G4 dendrimer complexes for topical targeted iontophoretic delivery of phosphorothioate oligonucleotides [150]. In vitro experiments revealed that anodal iontophoresis of ASO–dendrimer complexes considerably improved ASO skin penetration and cellular uptake, leading to a 46% inhibition of Bcl-2 expression compared to just 20% with passive delivery of free ASOs. Additionally, in vivo studies showed a gradual reduction in skin tumour size by up to 40% after a seven-day treatment with antisense dendriplexes while no significant changes were observed in mice treated with free ASOs. More recently, Tai et al. designed penetratin-conjugated PAMAM G5 dendrimers complexed with an ASO targeting luciferase expression in intraocular tumours [151]. The incorporation of the penetratin moiety significantly enhanced cellular uptake and gene silencing, while G5 dendrimers provided efficient ASO loading and maintained low toxicity. A similar strategy was used by Zhang and coworkers [152], who evaluated the efficacy of folic acid (FA)-conjugated PAMAM G5 dendrimers for delivering anti-EGFR ASOs to brain tumours. Gene knockdown and antitumoral essays using an intracranial glioma model demonstrated that FA-PAMAM vehicles provided greater inhibition of EGFR expression than PAMAM carriers and passive ASO delivery, which led to substantial tumour suppression two weeks after tumour implantation and prolonged survival. Subsequent studies, confirmed that low-generation PAMAM dendriplexes (G1–G5) could effectively cross the BBB and deliver antisense and siRNA oligonucleotides into brain tissue with minimal cytotoxicity, highlighting the immense potential of these platforms for antisense-based brain therapy [153–154]. In a related study, Xu et al. investigated the in vivo delivery of siRNA targeting VEGF using FA-decorated PAMAM G4 dendrimers in xenograft mice with head and neck squamous cell carcinomas [155]. These platforms enabled targeted antisense delivery to cancer cells, which diminished immune-related and toxic systemic adverse events. Moreover, whole-body fluorescence imaging demonstrated that G4-FA/siVEGF significantly inhibited tumour growth as well as reduced blood vessel formation in single-dose and two-dose regimen studies.

In order to enhance the treatment efficacy in drug-resistant tumours, Torchilin and coworkers developed hybrid lipid–polymer nanocarriers composed of PAMAM G4–*b*-PEG_2000_-*b*-DOPE copolymers encapsulated within PEG_5000_–PE micelles (DOPE = 1,2-dioleoylphosphatidyl ethanolamine and PE = phosphatidylethanolamine) for the co-delivery of anti-GFP siRNA and doxorubicin (DOX) [156]. The mixed micellar systems exhibited increased colloidal stability, greater ASO and drug loading, and enhanced siRNA/DOX internalisation than free modified dendrimers. Recent studies by Shi and coworkers have been focused on the development of multifunctional PAMAM G5 dendrimers entrapping metallic nanoparticles for the targeted delivery of anti-VEGF and anti-Bcl2 siRNAs [157–158]. In these studies, dendrimer-entrapped gold nanoparticles (Au-DENPs) were functionalised with arginine–glycine–aspartic acid (RDG) peptides and β-cyclodextrin, which have previously proven enhanced cell adhesion and greater efficiency compacting siRNA, respectively. These modified Au-DENPs exhibited high biocompatibility combined with outstanding transfection activity, leading to efficient silencing of glioblastoma cancer cells by downregulation of VEGF and Bcl2 proteins. In a separate study, G5 dendrimers entrapping MoS_2_ nanoflakes were evaluated for combinational gene silencing and photothermal therapy (PTT) in xenograft mice with breast cancer [159]. This synergistic effect was demonstrated by irradiating 4T1 cells and mice after intravenous administration of G5-MoS_2_/Bcl-2 siRNA polyplexes. In vitro and in vivo experiments revealed targeted intratumoral delivery of anti-siBcl2 along with a localised temperature increase in tumour tissue. This dual approach produced a significantly stronger antitumoral response, reducing cell viability to 21%, compared to 45.8% and 68.7% decrease achieved by single PTT therapy and G5-Bcl2 delivery, respectively.

**Poly(vinylamine).** In 2017, Dréan et al. first reported the potential of poly(vinylamine) (PVAm) as a non-viral vector for gene transfection [160]. Although the application of PVAm systems in antisense therapy has been significantly less explored, recent studies have demonstrated the suitability of PVAm for delivering antisense siRNA [161]. Tian et al. conducted a pioneering comparative study to evaluate the differences in toxicity and bioactivity of PVAm and PEI_20kDa_ systems conjugated with a variety of RNA cargoes [162]. Both platforms demonstrated high transfection activity and minimal immune-related effects in two xenograft mouse models with osteosarcoma and hyperlipidemia, leading to efficient antitumour activity and reductions in blood lipid levels, respectively. However, PVAm nanocomplexes outperformed their PEI counterparts during toxicity assessments in vitro, showing enhanced cell viability and lower hemolysis rates at all the concentrations for PVAm complexes, compared to PEI polyplexes, which exhibited notable dose-dependent toxicity. In a similar study, Lin et al. presented a novel arginine-modified PVAm system (PVAm-Arg) as ideal candidate for RNA oligonucleotide delivery [163]. In this report, PVAm-Arg–siRNA considerably improved siRNA condensation and delivery efficacy compared to commercial cationic liposomes (GP-siRNA-Mate Plus) and unmodified PVAm–siRNA polyplexes. In a later study, Qian and coworkers [164] evaluated the efficacy of peptide-conjugated PVAm–siRNA complexes to treat bone-related diseases. The presence of a serine–aspartate–serine–serine–aspartate peptide on the particle surface allowed for selective and enhanced delivery of anti-miR-138-5 oligonucleotides to osteoblasts, without affecting their capacity to bind nucleic acid derivatives nor the cell internalisation rates. As a result, mice treated with modified-PVAm vectors exhibited superior inhibition of specific mRNA and protein downregulation, leading to increased osteoblastic cell migration along with improved trabecular and cortical bone regeneration.

#### Poly(methacrylate/cyanoacrylate/acrylamide/methacrylamide)s

**Poly(methacrylate)s.***Poly(methyl methacrylate).* In 2009, Rimessi et al. explored the use of cationic poly(methyl methacrylate) (PMMA) core–shell nanoparticles for the delivery of a 2′-*O*-methyl-phosphorothioate (2′OMePS) antisense oligonucleotide, M23D, to restore dystrophin expression in a mouse model of Duchenne muscular dystrophy (DMD) [165]. The researchers designed PMMA nanoparticles (T1) with a cationic surface through emulsion polymerisation, which effectively bound ASOs and facilitated their systemic delivery. The study demonstrated that weekly intraperitoneal injections of these ASO-loaded T1 nanoparticles at a low dose (0.9 mg·kg^−1^ per week) led to significant restoration of dystrophin expression in skeletal muscles and the heart of the treated mdx mice, a commonly used model for DMD. Notably, the T1 nanoparticles were able to achieve this effect at a fraction of the dosage required when using naked ASOs, underscoring the efficiency of the PMMA-based delivery system. Building on this previous research into the use of PMMA for gene therapy, Ferlini et al. advanced the field by developing nanoparticles (ZM2) composed of a PMMA core and a *N*-isopropylacrylamide/2-(dimethyloctyl)ammonium ethyl methacrylate bromine copolymer shell, designed to deliver M23D ASO for the treatment of DMD [166]. Systemic administration of ZM2–ASO complexes effectively restored dystrophin protein expression in both skeletal and cardiac muscles of mdx mice. Notably, these ZM2 nanoparticles achieved significant dystrophin restoration in up to 40% of muscle fibres, with exon 23 skipping levels reaching up to 20% in skeletal muscles. These results highlighted the enhanced efficacy of PMMA-based nanoparticles in promoting dystrophin expression at lower doses of ASOs compared to previous methods. Alternatively, in their 2012 study, Bassi et al. demonstrated the long-term benefits of this approach [167]. Specifically, this report showed that the ZM2 nanoparticles not only facilitated significant dystrophin restoration in skeletal muscles but also maintained this expression for up to 90 days after administration, which was a marked improvement in durability compared to the earlier study. The later study reported persistent dystrophin expression in 7.2% of muscle fibres in the quadriceps and 4.5% in the diaphragm, highlighting the extended therapeutic potential of the ZM2 nanoparticles.

*Poly(methacrylic acid)/poly(acrylic acid) and derivatives.* The use of methacrylic and acrylic acid derivatives in drug and gene delivery has been explored in several studies, each demonstrating unique advantages. In 2013, Sevimli et al. focused on gene delivery applications using methacrylic acid derivatives, specifically poly(methacrylic acid-*co*-cholesteryl methacrylate) (P(MAA-*co*-CMA)), and quaternized poly((dimethylamino)ethyl methacrylate-*co*-cholesteryl methacrylate) (Q-P(DMAEMA-*co*-CMA)) with varying ratios of cholesterol methacrylate content (2–20%) [168]. In order for the anionic MMA-containing monomer to form stable complexes with negatively charged, anti-enhanced green fluorescence protein (anti-GFP) siRNA, a cationic oligolysine (DP = 10) linker, was introduced into the mixture. While P(MAA-*co*-CMA) exhibited low cytotoxicity, its transfection efficiency was limited because of poor cellular internalisation. However, the Q-P(DMAEMA-*co*-CMA) complexes, particularly at an N/P ratio of 20:1, showed much higher gene silencing efficiency. The Q-2% CMA variant achieved the greatest suppression of GFP expression, indicating the superior gene silencing potential of methacrylic acid derivatives when tuned for siRNA delivery, especially due to their enhanced endosomal escape. In a separate research, Shrestha et al. investigated the targeted delivery, transfection efficiency, and detection capabilities of multifunctional poly(acrylic acid)-*b*-poly(octadecyl acrylate-*co*-decyl acrylate) (PAA-*b*-PODA-*co*-PDA)) cross-linked nanoparticles complexed with antisense PNAs to diagnose and treat acute respiratory distress syndrome [169]. PNA nanoparticles modified with cell penetrating peptides to facilitate cellular entry and a ^64^Cu radionucleotide for imaging purposes demonstrated selective binding to their specific mRNA while maintaining optical biostability. Early studies by Lee et al. explored the effects of incorporating poly(propylacrylic acid) (PPAA), a pH-sensitive and membrane-disruptive polyanion, into cationic 1,2-dioleoyl-3-trimethylammonium propane (DOTAP)–ASO formulations [170]. In vitro studies demonstrated that DOTAP–PPAA–ASO complexes (PPAA, *M*_n_ = 27 kDa) improved the internalisation of phosphorothioate oligonucleotides as well as their intracellular release, which resulted in superior inhibition of d1EGFP expression compared to DOTAP–ODN complexes. Similarly, Peddada et al. explored acrylic acid derivatives in the form of PPAA (*M*_n_ = 200 kDa) for the delivery of the Bcl2 antisense oligonucleotide (5′-TCTCCCAGCGTGCGCCAT-3′) for the potential treatment of ovarian cancer [171]. By optimising the HLB of PPAA–poly(alkylene oxide) graft copolymers, they improved membrane penetration and gene silencing efficiency. The PPAA copolymers, especially those with lower HLB values, led to up to a 60% reduction in Bcl-2 gene expression in A2780 ovarian cancer cells. Additionally, in vivo studies demonstrated better biodistribution and accumulation of ASOs in tumour xenografts, with greater stability and efficacy compared to traditional DOTAP–ASO complexes.

*Poly((dialkylamino)ethyl methacrylate).* Building on the promising research conducted by Ferlini and Rimessi et al., who utilised 2-(dimethylamino)ethyl methacrylate (DMAEMA)-based polymers for gene therapy [165–166], Beavers et al. developed porous silicon–polymer nanocomposites incorporating poly[(ethylene glycol)-*b*-(2-(dimethylamino)ethyl methacrylate-*co*-butyl methacrylate)] (PEGDB) for the delivery of a cysteine-modified peptide nucleic acid (NH_2_-ACAAACACCATTGTCACACTCCA-Cys-COOH) as an anti-miRNA (miR122) therapy [172]. The study demonstrated that the inclusion of DMAEMA in the polymer matrix significantly enhanced endosomal escape, leading to improved cytosolic delivery and bioactivity of the PNA. This polymer was finely tuned to disrupt membranes in acidic environments, such as those found in endosomes, but to remain stable at physiological pH. The research showed that these nanocomposites exhibited increased circulation half-life and bioavailability of PNAs in vivo, resulting in effective inhibition of miR-122 in a mouse model. Grimme et al. further explored the potential of DMAEMA in forming polycationic micelleplexes specifically designed for the delivery of short ASOs [173]. This study compared various polymer architectures, including linear homopolymers and amphiphilic di- and triblock copolymers incorporating DMAEMA, in terms of their effectiveness in complexation and delivery of a 16 bp antisense oligonucleotide, ENZ-4176. Micelleplexes constructed from poly(ethylene glycol)methylether methacrylate (PEGMEMA_20_, *M*_n_ ≈ 500 Da, O_b_), DMAEMA_160_ (D), and butyl methacrylate (BMA_92_, B) triblock copolymers (O_b_DB) demonstrated significantly higher stability compared to their diblock (O_b_D and DB) and homopolymer (D) polyplex counterparts because of their dense PEG corona. However, reduced cellular internalisation was observed compared to the DB micelleplex (ca. threefold), as a consequence of the PEG corona. Among the different formulations, the DB diblock micelleplexes achieved the most pronounced results, achieving up to 79% gene silencing efficiency. This work underscored the critical role of polymer architecture in enhancing nucleic acid delivery and highlighted DMAEMA-based micelleplexes as a promising approach for improving the efficacy and consistency of ASO therapies. More recently, Mohamed et al. utilised DMAEMA as a key component in a novel pH-responsive block copolymer system designed for the co-delivery of DOX and antisense oligonucleotide GTI2040 to MCF-7 breast cancer cells [174]. The researchers synthesised a triblock copolymer, PEG_44_-*b*-PDMAEMA_39_-*b*-PHEMA_60_, and conjugated DOX through pH-sensitive imine linkages to the PHEMA block, forming cationic self-assembled nanoparticles (DOX-SAN). The nanoparticles were further complexed with negatively charged GTI2040, forming stable nanocomplexes that demonstrated significant pH sensitivity, with enhanced DOX release at acidic pH levels. Notably, the GTI2040/DOX-SAN system exhibited a hydrodynamic size of 136.4 nm and a zeta potential of 21.0 mV, and provided superior cellular uptake and gene silencing efficiency compared to free GTI2040 and DOX alone. The co-delivery system outperformed single-agent therapies in inhibiting the R2 protein of ribonucleotide reductase, highlighting a potential novel platform combination cancer therapy.

**Figure 10:**
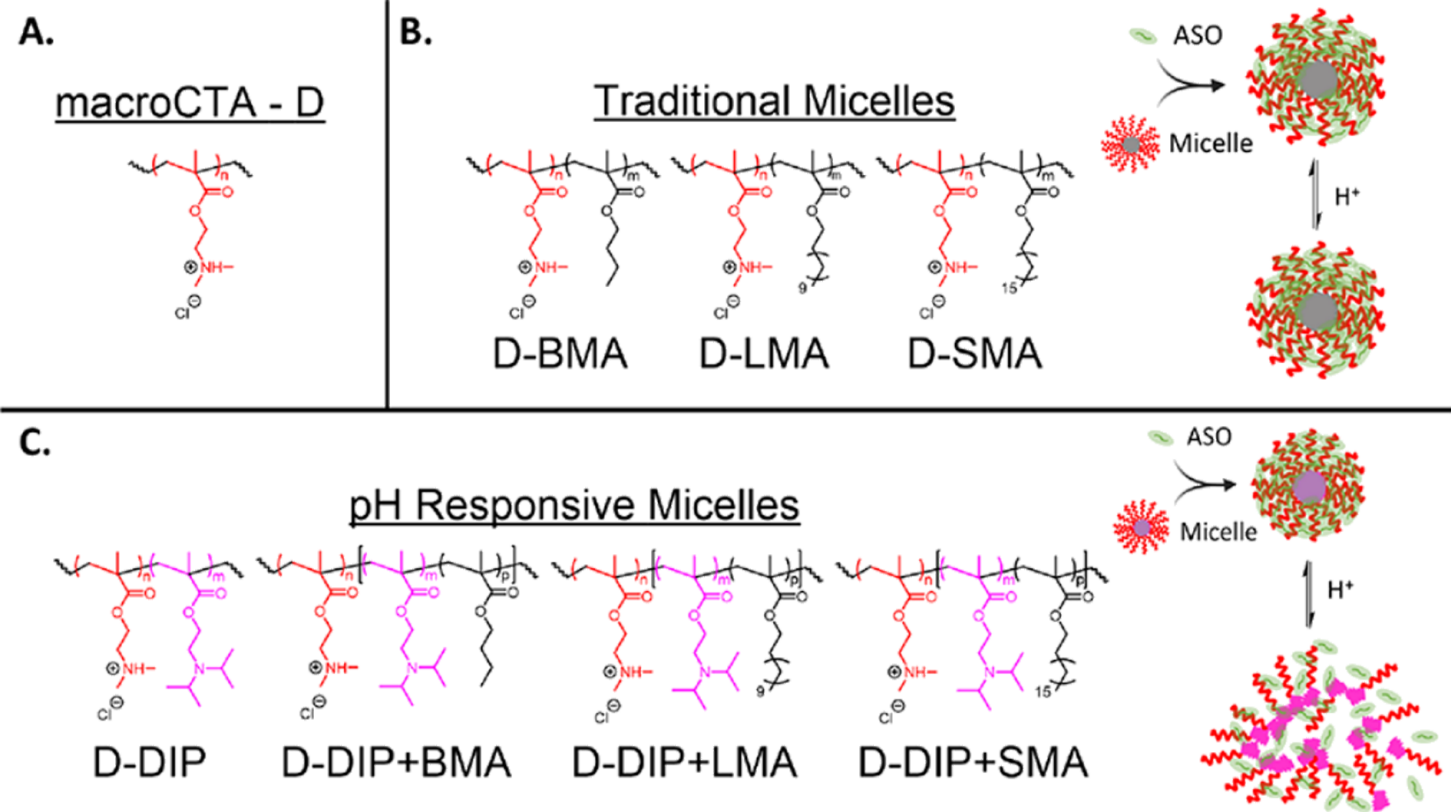
(A) Depiction of the cationic homopolymer D. (B) Depiction of the polymers for traditional micelles and a scheme to show the binding with ASOs and the effect of acidification. (C) Depiction of the polymers for pH-responsive micelles and a depiction of binding with ASOs and the effect of acidification. Figure 10 was adapted with permission from [175], Copyright 2023 American Chemical Society. This content is not subject to CC BY 4.0.

In their 2023 study, Grimme et al. utilised 2-(diisopropylamino)ethyl methacrylate (DIP) to design lipophilic, pH-responsive micelles for ASO delivery (Figure 10) [175]. The study compared DIP-containing micelles with varying alkyl chain lengths to assess their gene silencing efficiency. Key findings showed that micelles incorporating DIP and long alkyl chains, such as lauryl (D-DIP+LMA) and stearyl (D-DIP+SMA) methacrylate, achieved up to 90% gene silencing, outperforming shorter chain variants and non-pH-responsive micelles. Notably, these micelles demonstrated similar silencing efficacy to industry standards such as JET-PEI and Lipofectamine 2000, while exhibiting lower toxicity than Lipofectamine 2000. This underscored the importance of including the pH-responsive DIP unit to facilitate micelle disassembly and improve gene silencing efficiency, particularly in systems with longer alkyl chains.

**Poly(cyanoacrylate)s.** Cyanoacrylate-based polymers have long been recognized for their adhesive properties and are widely used in medical applications such as tissue adhesives and wound closure [176–178]. More recently, their utility has expanded into drug and gene delivery systems, where their ability to form biodegradable nanoparticles has shown promise in targeted therapies [179–181]. These polymers, such as poly(butyl cyanoacrylate) (PBCA), are particularly valued for their capacity to cross biological barriers, such as the BBB, making them ideal candidates for delivering therapeutic agents to difficult-to-reach areas such as the brain. In this context, Taghavi et al. utilised PBCA nanoparticles to investigate their effects on the regulation of Bcl-2 family genes in glioblastoma cells [182]. The study aimed to assess the potential of PBCA nanoparticles in modulating apoptosis-related pathways without inducing cytotoxic effects. The researchers treated two glioblastoma cell lines (U87MG and A172) with PBCA nanoparticles (Z-average ≈ 172 nm) and found no significant impact on cell viability or induction of apoptosis or necrosis, as confirmed by flow cytometry analysis. However, the gene expression analysis revealed that PBCA nanoparticles significantly altered the expression of several Bcl-2 family members, including downregulation of Bcl-2L12 and upregulation of Bcl-XL in U87MG cells. These findings suggest that while PBCA nanoparticles did not induce immediate cytotoxicity, they may modulate apoptosis-related pathways, offering insights into their potential for targeted cancer therapy without harming normal cells. In 2021, Liu et al. explored the use of alkoxy cyanoacrylate-based nanoparticles for the targeted delivery ASOs to glioblastoma cells [183]. The researchers developed two novel nanoparticles, NP2 and NP3, based on 2-methoxyethyl cyanoacrylate and 2-(2-methoxyethoxy)ethyl cyanoacrylate, respectively. These nanoparticles were further coated with polysorbate 80 to enhance their ability to cross the BBB. The study demonstrated that NP3 exhibited superior stealth properties, significantly reducing phagocytosis by mononuclear macrophages while enhancing cellular uptake in glioma cells. In vitro and in vivo experiments revealed that NP3 effectively delivered ASOs to the brain, resulting in a marked reduction in TGF-β2 mRNA and protein expression in U251 glioma cells. This nanoparticle system showed promising anti-cancer effects and extended circulation time, highlighting its potential for targeted brain therapies.

**Poly(acrylamide)s and poly(methacrylamide)s.** Pioneering reports on poly(acrylamide)-based [184] and poly(methacrylamide)-based [185] platforms to deliver antisense PNAs and disulfide oligonucleotides, respectively, date back to the late 1990s. Since then, the field has grown significantly owing to the development of controlled radical polymerisation techniques, which has enabled the development of a wide range of acrylamide- and polyacrylamide-containing vectors for the safe and targeted delivery of ASOs, including cationic polymer complexes and cross-linked nanoparticle systems [186]. For example, Dautzenberg et al. demonstrated that high-molecular-weight PLL–ODN complexes (PLL, *M*_w_ = 13.4 kDa) grafted with short poly(*N*-(2-hydroxypropyl)methacrylamide) chains (PHPMA, *M*_w_ = 7 kDa) exhibited superior stability and reduced cytotoxicity under physiological conditions compared to non-modified PLL and poly(trimethylammonioethyl methacrylate chloride) [187].

Wooley and coworkers have done extensive research on the synthesis of cationic poly(acrylamide)-based shell-cross-linked knedel-like (cSCK) nanoparticles for antisense and gene therapy [188–189]. Initial work employed poly(styrene)-*b*-poly(acrylic acid) (PS-*b*-PAA) copolymers to prepare polymer micelles stabilised by subsequent cross-linking through carbodiimide coupling, to yield PNA-derivatised PS-*b*-poly(acrylamidoethylamine) (PAEA) cSCK nanoparticles [190]. Delivery of antisense PNA into cells could be achieved either through electrostatic complexation or reversible disulfide linkage between a PNA oligonucleotide hybrid (H-CCTCTTACCTCAGTTACA-NH_2_) and the cSCK nanoparticle. Lower toxicity was observed for cSCK particles compared to PNA-complexed lipofectamine and arginine systems. Moreover, cross-linked nanoparticles were found to facilitate both PNA endocytosis and endosomal escape in HeLa cells, resulting in superior switch splicing activity and luciferase regulation [191]. Later studies have employed this strategy to design novel PAEA-*b*-PS platforms to diagnose acute lung injuries at early stages through the delivery of hybrid PNAs containing a tyrosine residue for radiolabelling and an arginine peptide to facilitate oligonucleotide endosomal and lysosomal escape, targeting inducible nitric oxide synthase (iNOS) mRNA [192]. In vitro and in vivo studies showed selective retention of PNA in mouse lung, accompanied by a marked reduction in iNOS-associated inflammation. These findings highlight the potential of cSCK nanoparticles as antisense delivery vectors, offering promise for both diagnostic and therapeutic applications.

Recently, Reineke and coworkers investigated the role of chemical composition and polymer architecture for the delivery of anti-deGFP ASOs to human embryonic kidney cells [193]. In this study, ASO-conjugated homopolymers and diblock copolymers of (aminoethyl)acrylamide (AEA), (dimethylaminoethyl)acrylamide (DMA), (diethylaminoethyl)acrylamide (DEA), (trimethylaminoethyl)acrylamide (TMA), and (morpholinoaminoethyl)acrylamide (MEA) were used to prepare cationic polyplexes and micelleplexes. Micelle–ASO complexes, owing to their higher colloidal stability, outperformed their linear analogues in both cell viability and GFP knockdown assays. Interestingly, the cellular uptake and transfection efficiency were found to be strongly influenced by the degree of amine substitution and bulkiness of the cationic moiety. Complexes containing bulkier and/or tertiary amine groups exhibited significantly greater ASO internalisation. However, polymers with primary amines and smaller moieties showed more efficient ASO transfection (AEA > DMA > DEA > TMA > MEA), suggesting that internalisation alone is not the primary factor determining bioactivity. Subsequent studies analysed the ASO delivery capability of polymeric micelles composed of an *n*-butyl acrylate segment extended with AEA, DEA, and MEA (referred to as A, D, and M, respectively), mixed micelles involving two types of micelles (A+M and D+M), as well as blended micelles comprising a combination of A/M and D/M copolymers [194]. It was observed that mixed and blended systems, particularly D and M combinations with low D incorporation (D20M80), boosted the ASO transfection efficiency compared to homomicelles, while still maintaining high biocompatibility.

#### Neutral polymers

Cationic polymers have proven highly efficient in the delivery of nucleic acids. However, positively charged polyplexes usually exhibit low stability in plasma and high levels of toxicity depending on their composition, molecular weight, architecture, and charge ratio, which hinder their clinical application [186]. Alternatively, the design of delivery systems using neutral polymers has arisen as an attractive strategy to overcome these limitations. Even though the use of neutral polymers for antisense therapy has been less explored than that of their cationic counterparts, they have shown to offer improved colloidal stability, reduced aggregation to serum proteins and immunogenicity, which prevent non-specific uptake and encourage specific cellular targeting [144,195–196]. This section explores the incorporation of neutral polymers in ASO-based delivery systems and their pharmacokinetic impact.

**Poly(ethylene glycol).** PEG is a biocompatible, highly flexible, water-soluble neutral polymer bearing hydroxy end groups that allow for post-polymerisation functionalisation. For many years, the chemical conjugation of PEG to antisense RNAs, as well as its incorporation in lipid and polymer-based formulations has been studied to address the unfavourable absorption, distribution, metabolism, excretion, and toxicity (ADMET) properties usually associated with free drug administration [197–198]. In late 2004, the U.S. Food and Drug Administration (FDA) recognised the safety of PEG and approved the use of Macugen^®^, the first PEG–RNA oligonucleotide system, for the treatment of neovascular age-related macular degeneration (AMD) [199]. Since then, the application of PEG has widely expanded allowing for the development of a broad range of platforms for gene delivery [200].

PEGylation affords systems with a hydrophilic non-ionic inert corona that protect the conjugated drug through the formation of a hydration shell, which inhibits proteins and other biomacromolecules from binding to the drug and, therefore, enhances the colloidal stability and reduce aggregation and immunogenicity [201]. For example, Baek et al. studied the critical role of PEG on the pharmacokinetic properties and toxicity of PNA adsorbed-graphene oxide (nGO) nanosized systems for antisense therapy [202]. PEG–nGO carriers (six-arm PEG, *M*_w_= 15 kDa) exhibited excellent biocompatibility even at the highest concentrations compared to PNA–nGO (90% vs 50% cell viability at 100 μg/mL). Moreover, while PNA internalisation was not observed for the free-PNA and PNA–nGO groups, strong cytoplasmatic fluorescence was detected with the PEGylated group, which indicated significant cellular PNA uptake and, consequently, optimal gene knockdown. Moreover, this characteristic “shielding effect” prolongs the circulation time of conjugated oligonucleotides since renal excretion is effectively reduced, which directly affects their biodistribution and cell uptake [203].

Numerous reports have shown that the success of PEG-based systems is highly influenced by composition, polymer architecture, molecular weight *M*_w_, and grafting density [204–206]. For instance, Miteva et al. demonstrated that length and density of PEG chains enhanced cytoplasmatic bioavailability of siRNA and gene silencing, despite a lower cellular internalisation rate [207]. In this study, copolymers of PDMAEMA-*b*-(DMAEMA-*co*-PAA-*co*-BMA) and PEG-*b*-(DMAEMA-*co*-PAA-*co*-BMA) with PEG molecular weights ranging from 5 to 20 kDa were prepared and assembled into mixed micelles, which were loaded with an anti-luciferase siRNA. Both in vitro and in vivo studies revealed that PEG-coated micelles with higher molecular weights exhibited greater efficiency of intracellular RNA unpacking and endosomal escape than non-coated and lower-molecular-weight PEG micelles, resulting in improved intracellular bioavailability and gene knockdown. In addition, Vinogradov et al. reported the physical properties and biostability of different polymeric complexes from PEG8000–polyamine block copolymers after the conjugation of a phosphorothioate oligonucleotide (PS-ODN) and observed that PS-ODN/PEG-*b*-PEI complexes (PEI, *M*_w_ = 2000 g·mol^−1^) were more stable in serum proteins and larger in size compared to PS-ODN/PEG-*b*-PSP complexes (32 nm vs 12 nm; PSP = polyspermine) [208]. Moreover, Park and coworkers explored the synthesis of hybrid PEG–ASO-conjugated micelles with multiple cationic polymers, peptides, and hydrophobic moieties, including PEI, PLL, protamine, fusogenic KALA peptide, and folate, and examined their intracellular uptake, systemic distribution, and pharmacological effects [117,209–210]. In other studies, Discher and coworkers investigated the use of biodegradable polymersomes for delivering ASOs and siRNA to muscle tissue [211–212]. Polymersomes of PEG_52_-*b*-PCL_44_ and PEG_15_-*b*-PLA_70_, blended with inert PEG-*b*-butadiene, were synthesised to achieve controlled and predictable release of both gapmer ASOs and ssASOs while exhibiting high cytocompatibility and cellular uptake by muscle cells, which resulted in efficient regulation of dystrophin.

The impact of polymer architecture and particle morphology on effective drug delivery is often overlooked, despite its significant role in overcoming biological barriers [213–214]. Recent advances have shown that complex PEG structures such as dendrimers, star, and bottle-brush polymers helped to reduce the spacing between polymer chains, leading to higher steric shielding and more efficient biodistribution of the cargo, and reduced toxicity [215]. In this regard, Wang et al. reported a densely packed PEG bottle-brush carrier, bearing ca. 30 PEG side chains (10 kDa each), containing covalently bound RNA oligonucleotides [216]. These carriers exhibited superior circulation lifetimes and drug plasma availability compared to unmodified RNA systems. As a result, this approach led to reduced hepatotoxicity, increased tumour uptake, and enhanced gene silencing. Qi and coworkers further explored the effect of PEGylation and polymer architecture by designing PEGylated PAMAM dendrimers (PEG, *M*_w_ = 5 kDa) loaded with siRNA [217]. Biological studies showed enhanced solubility and gene regulation, along with reduced immunogenicity and hepatic toxicity, emphasising the crucial role of polymer design and PEGylation in effective nucleic acid delivery.

**Figure 11:**
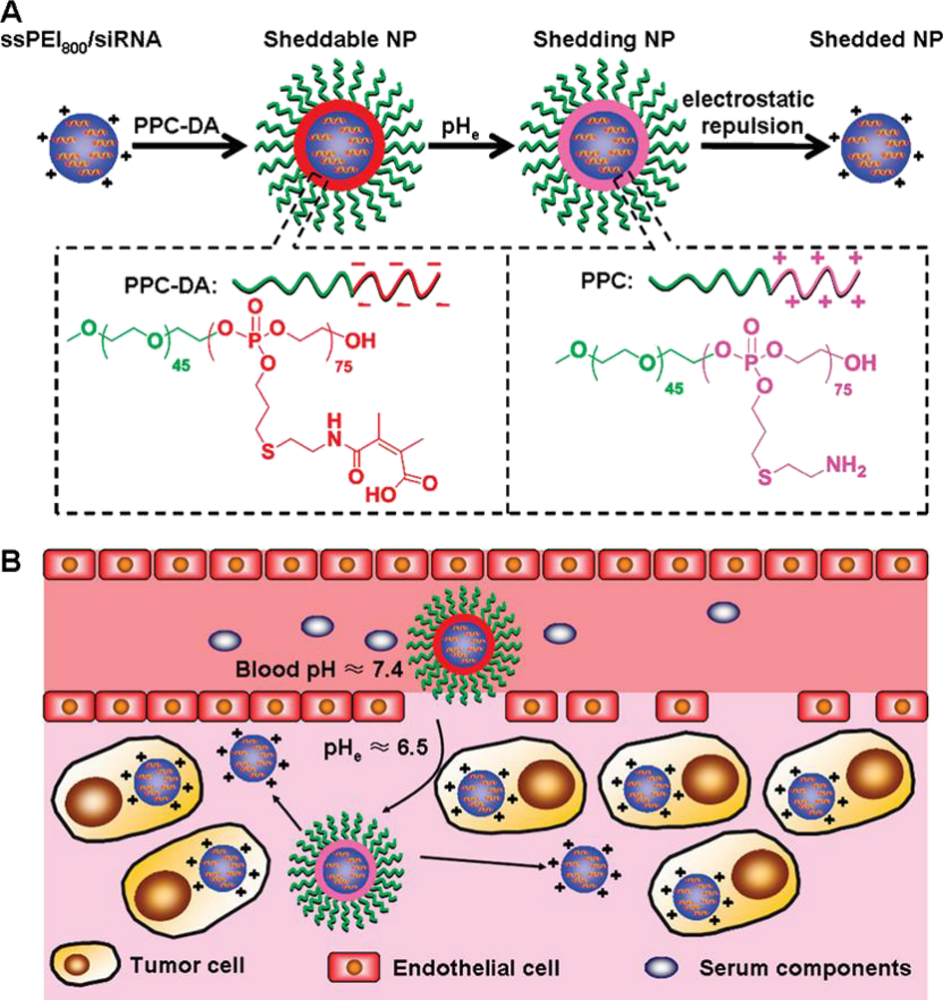
pH-responsive PEGylated systems for antisense oligonucleotide delivery. Figure 11 was reprinted with permission from [222], Copyright 2012 American Chemical Society. This content is not subject to CC BY 4.0.

Even though PEGylation of cationic polymer–ASO delivery vectors have proved advantageous tackling many of the challenges associated with in vivo delivery of cationic vehicles, the subsequent decrease in zeta potential of these platforms results in significantly lower cellular internalisation rates. As an attempt to overcome this limitation, many groups have focused on designing responsive PEGylated systems, where PEG chains are cleaved from delivery systems in response to environmental triggers [218]. For instance, Li et al. developed enzyme-responsive PEGylated polyplex micelles through the incorporation of a peptide linkage, between the PEG and poly(aspartame) segments, susceptible to metalloproteinases (MMPs), which are highly expressed in tumour tissues [219]. Consequently, in presence of MMP-2, the PEG_227_ block was cleaved, exposing the micelles’ positively charged surface, which resulted in enhanced cellular internalisation and efficient endosomal escape. The design of delivery systems responsive to changes under acidic conditions have attracted considerable attention owing to the different pH present in physiological and damaged tissues [220]. Oishi et al. reported the synthesis of PLL micelles complexed with lactosylated PEG–ASO conjugates (allyl-PEG-OH, *M*_n_ = 4340 Da) containing an acid-labile β-thiopropionate linkage between PEG and ASO segments for targeted intracellular delivery [221]. The lactosylated polymeric micelles showed improved association with HuH-7 cells and a pronounced antisense effect. Later studies by Wang and coworkers investigated the synthesis and applicability of PEGylated ternary particles for the tumoral delivery of antisense RNA [222]. At physiological conditions (pH 7.4), these particles were designed to display anionic and cationic groups, which neutralised the particle surface and reduced the interaction with serum proteins. Whereas, upon arrival to the tumour microenvironment (pH 6.4), a transition from negatively to positively charged groups followed by PEG shedding enabled efficient RNA delivery into cancer cells and enhanced gene silencing (Figure 11).

Despite the obvious advantages of PEGylation, recent evidence also showed that the incorporation of PEG chains can trigger two potential immune responses, which should be taken into consideration before designing PEGylated systems. The first one is a chronic immunogenic response, which involves the formation of PEG-specific antibodies and leads to accelerated blood clearance rates of PEGylated vectors [223]. The second is an acute complement activation-related pseudo-allergenic response and hypersensitivity reactions against PEG-based materials [224]. For this reason, alternative polymers for coating nanoparticles including natural polysaccharides, polypeptides, and other stealth polymers have been studied [225].

**Poly(lactide-*co*-glycolic acid).** PLGA is a non-toxic and biodegradable polyester made from a combination of lactide and glycolic acid esters. Owing to its robust thermo-mechanical properties and high biocompatibility, PLGA has been extensively studied for a broad range of biomedical applications, such as drug and nucleic acid delivery systems [226–227], scaffolds for tissue engineering [228], enhanced imaging [229], and immunotherapy [230]. In addition, the polymer’s characteristic degradation profile and its suitability for post-polymerisation functionalisation have enabled the development of PLGA-based platforms for controlled release of siRNA and ASOs. Multiple reports by De Rosa et al. investigated the encapsulation efficiency and the release profile of several PLGA microsphere formulations loaded with free ODNs and PEI/ODN complexes (branched PEI, *M*_w_ = 25 kDa) with varying N/P and lactide/glycolide (LA/GA) ratios [231–234]. In these studies, both N/P ratio and PEI content were found to play crucial roles regarding the particle morphology and the release profile. Larger microspheres were obtained with increasing N/P ratios and higher PEI incorporation compared to ODN–PLGA particles with lower N/P ratios, leading to improved encapsulation efficiency. However, PEI/pdT16 complexed vehicles displayed larger pore diameters, causing a rapid release of ODNs, where 80% of the content was released within the first four days. To reduce this burst effect and achieve a more controlled, sustained release, additives were introduced. Formulations of PLGA-PEI/pdT16 containing 3% of sodium chloride displayed smaller pore sizes and enabled a more homogenous ODN distribution within the polymeric matrix, which led to a significant reduced burst effect accompanied with slower release rates, while maintaining low cytotoxicity, enhanced intracellular penetration, and resistance to enzymatic degradation. The LA/GA ratio was also shown to significantly influence the release rates of the system [233]. While microspheres composed by a 50:50 ratio exhibited an uncontrolled release profile, characterised by a substantial burst effect with 80% of the content being immediately released, particles formulated with a 75:25 ratio of LA/GA showed a sustained drug release over time with a very low initial burst effect.

These platforms have been investigated for targeted ASO delivery in several tissues, Carrasquillo et al. reported the encapsulation of the previously discussed PEGylated oligonucleotide pegapnatib into PLGA (50:50) microspheres for successful transscleral delivery to treat ocular angiogenesis [235]. In another study, with the aim to prevent subconjunctival fibrosis after glaucoma filtering surgery, Fattal and coworkers designed porous PLGA microspheres (PLGA 75:25, *M*_w_ = 98.9 kDa) containing free anti-TGF-β2 antisense phosphorothioate oligonucleotides and PEI–phosphorothioate oligonucleotide complexes (branched PEI, *M*_w_ = 25 kDa) [236]. During in vitro and in vivo studies, these biodegradable microparticles demonstrated advantageous at delivering oligonucleotides to cellular nuclei while providing a sustained release over extended periods, which could be controlled by tailoring the pore size. Tahara et al. designed chitosan-functionalised PLGA nanospheres (PLGA 75:25, *M*_w_ = 20 kDa) to deliver a nuclear factor kappa B (NF-κB) oligonucleotide to the gastrointestinal tract [237]. These chitosan–PLGA nanocarriers provided superior uptake of ODNs in Caco-2 cell monolayers compared to non-functionalised PLGA complexes, leading to enhanced anti-inflammatory response and increased myeloperoxidase activity. As a result, significant improvements in ulcerative colitis symptoms were observed during in vivo analysis following oral administration. Moreover, the use of PLGA-based systems has been widely studied regarding lung-targeted diseases. Taez et al. studied the efficacy of cationic chitosan–PLGA (70:30) nanoparticles loaded with antisense 2′-*O*-methyl-oligonucleotides targeting telomerase activity in several lung cancer cell lines [238]. In this study, ASO-conjugated PLGA particles bearing chitosan moieties on the surface exhibited optimal stability and enhanced cellular internalisation, even after the neutralisation of positive charges at physiological pH. Consequently, significant telomere shortening was observed, and telomerase activity was strongly inhibited across all cancer cell lines. Moreover, Saltzman and coworkers explored the stability, cellular uptake, in vivo biodistribution, and therapeutic efficacy of antisense nanoparticles made from a blend of PLGA and poly(β-amino ester) to deliver triplex-forming PNAs and DNA to transgenic mice with cystic fibrosis [239–241]. In 2018, Ricciardi et al. pioneered the in utero delivery of PLGA_(50:50)_–(PNA/DNA) complexes to foetal tissues for gene and protein modulation, without affecting the prenatal development nor compromising the adult fertility capacity [242].

Antisense PLGA platforms have shown great potential for the development of novel targeted delivery systems for cancer therapy. McNeer et al. investigated the use of PLGA nanoparticles to simultaneously deliver triplex-forming PNAs and DNA to primary CD34^+^ human hematopoietic progenitors, which are highly involved in leukaemia and lymphoma [243]. Glazer and coworkers designed a novel therapeutic approach using PLGA nanoparticles loaded with miniPEG-γPNAs targeted to oncogenic microRNAs (miR) [244]. Nanocarriers showed improved PNA solubility, high stability at physiological conditions, and excellent biocompatibility in vitro and during histopathologic essays, which in combination with the superior RNA binding affinity of γPNA oligonucleotides resulted in significantly lower cell proliferation rates and tumour growth compared to control experiments. In another study, the same technology was employed to knockdown the chemokine receptor 5 (CCR5), a cell membrane receptor required by HI virus to infect the host CD4^+^ T cells [245]. Here, ^MP^γPNA-treated cells showed a 40% decrease in CCR5 mRNA levels after 24 h, which led to subsequent reduction of CCR5 protein expression. Alternatively, Bozkir and coworkers developed lipid–polymer systems for dual-targeted delivery of anticancer drugs and double-stranded anti-miR-210 ASOs to the brain for glioblastoma treatment [246]. Hybrid nanoparticles were prepared through a double-emulsion method using PLGA (50:50) and a phosphatidylcholine/trimyristin mixture as the polymer and lipid phases, respectively. Incorporating 10 wt % poly(caprolactone) into the formulation improved the drug loading efficiency, while ensuring outstanding stability and a sustained, slow release of therapeutic agents under physiological conditions. Additionally, cytotoxicity studies on grade-IV glioblastoma cells demonstrated considerable cell growth inhibition. These findings underscore the significant therapeutic potential of PLGA-based systems.

**Poly(ε-caprolactone).** Poly(ε-caprolactone) (PCL) is another polyester that has recently garnered plenty of attention in the biomedical field owing to its high biocompatibility, robust thermal and mechanical properties, and slow degradation rates. Xiong et al. prepared a series of PCL-*b*-PEG block copolymers (PEG, *M*_n_ = 5000 g·mol^−1^) grafted with polycationic side chains of spermine, tetraethylenepentamine, and *N*,*N*-dimethyldipropylenetriamine, which were able to bind siRNA oligonucleotides [247]. PCL-based micelles were successful at protecting the antisense cargo from degradation and showed efficient cellular uptake by MDA435/LCC6 cells transfected with MDR-1, leading to significant reduction of P-glycoprotein expression. In recent reports, novel star-shaped PCL-*b*-PEG nanoparticle systems containing glucose molecules and anti-nucleolin aptamers conjugated to the semicrystalline core block and PEG corona, respectively, were designed for the targeted delivery of both cisplatin (CIS) and anti-miR-214 LNAs to CIS-resistant ovarian tumours [248]. In vitro and in vivo studies demonstrated that these platforms effectively released CIS and LNA in a controlled and sustained manner for 72 h, following initial burst releases of 42% and 49%, respectively. As a result, significant inhibition of endogenous miR-214 and enhanced drug sensitivity in CIS-resistant cell lines were observed, leading to increased necrosis and apoptosis rates compared to naked or non-aptamer transfected systems. In another study, Wang and Sun reported the preparation of a series of hybrid polymeric micelles for the co-delivery of anti-NF-κB siRNAs and the small-molecule drug dexamethasone [249]. By adjusting the ratio of cationic PCL-*b*-PEI and PCL-*b*-PEG, carriers with various physicochemical properties and drug concentrations were obtained. In vitro and in vivo studies demonstrated the ability of the systems to enhance suppression of NF-κB signalling and improved anti-inflammatory response, which underscored the potential of this synergistic approach for treating rheumatoid arthritis.

The fabrication of cell scaffolds for tissue engineering has emerged as a promising strategy for regenerative medicine. Among all polymers, PCL has raised great attention because of the high biocompatibility and semicrystalline nature, which enable the synthesis of robust and non-toxic platforms for long-term therapeutic use. In this regard, Cao et al. explored an alternative delivery approach to locally deliver double-stranded ASOs through a scaffold-mediated system [250]. In this work, siRNA oligonucleotides targeted to suppress glyceraldehyde 3-phosphate dehydrogenase (GAPDH) mRNA expression were adsorbed into PCL nanofibers with and without PEG obtained by electrospinning. Controlled and sustained delivery to human embryonic kidney cells and mice fibroblasts was achieved for 28 days under physiological conditions, leading to enhanced cellular uptake and higher mRNA downregulation efficiency compared to passive siRNA delivery systems. It is worth mentioning that PCL–PEG systems (with *M*_w_(PCL) = 65000 g·mol^−1^ and *M*_w_(PEG) = 3350 g·mol^−1^) showed significantly higher encapsulation and release efficiencies than PCL fibres, with 26% and 3% of siRNA released, respectively, with respect to the initially loaded amount. Despite vast differences in release efficiency, very similar GAPDH silencing was observed for all types of nanofibers. These results open a new pathway to design local RNA–ODN delivery platforms, which provide a suitable environment for tissue regeneration as well as the ability to modulate cellular response with antisense therapy.

**Other polymers.** Similar to PLGA and PEG, other biodegradable and stealth polymeric matrices have been studied to enhance the delivery of ASOs and nucleic acids. For example, the use of poly(lactide) (PLA) films for sustained delivery of antisense phosphodiester and phosphorothioate oligonucleotides was first investigated in 1995 by Akhtar and coworkers [251]. In the early 2000s, Delie et al. explored the encapsulation, release profile, and biocompatibility of PLA particles (*M*_w_ = 100 kD) loaded with phosphorothioate oligonucleotides [252]. In this study, PLA-based micro- and nanoparticles exhibited moderate encapsulation efficiencies and diverse release profiles, depending on the size and assembly methodology. Later studies reported the development of antibody-targeted micelles composed by poly(lactide)_130_-*co*-poly(2-methyl-2-carboxy trimethylene carbonate)_10_-*graft*-poly(ethylene glycol)_227_-azide (P(LA-*co*-MTCC)-*g*-PEG-azide, PEG = 10 kDa), to which the monoclonal antibody trastuzumab and a combination of 2′-modified ASOs and phosphorothioate ASOs were conjugated to the hydrophilic stealth corona via copper-mediated click addition [253]. In vitro studies showed that nanoparticles containing the three therapeutic agents induced prolonged luciferase silencing while keeping good bioavailability and low toxicity.

Other classes of biodegradable polymers that have been used in the biomedical field as matrices for delivering nucleic acid oligonucleotides include poly(phosphazenes), polypeptides and polypeptoids [254–255]. Even though the clinical application of poly(phosphazene)-containing systems has remained relatively unexplored as a consequence of their more complex synthesis and lower transfection efficiency compared to other studied polymers [256], some studies have demonstrated their potential in the pharmaceutical industry. Mutwiri et al. investigated the use of ASO-conjugated polyphosphazene polyelectrolytes as vaccine adjuvants to enhance the immunomodulatory response by delivering CpG oligodeoxynucleotides [257]. Whereas, Qiu and coworkers reported a novel composite poly(phosphazene) nanosized vesicle system to efficiently co-deliver miRNAs and placitaxel into drug-resistant lung cancer cells [258]. Also, polypeptides and polypeptoids (i.e., *N*-substituted amino acid derivatives, such as poly(sarcosine)) have recently emerged as promising alternatives to replace PEG [259–260]. These materials have demonstrated stealthiness and improved cellular uptake of mRNAs, resulting in enhanced transfection efficiency and protein regulation without inducing hepatotoxicity or immune responses [261]. However, to the best of our knowledge there are no previous reports focused on antisense oligonucleotides delivery using these platforms yet.

### Future perspectives

Antisense therapy has shown significant potential for tackling both rare and common diseases [262]. Rapid advances in nanotechnology and molecular science have allowed for the development of a wide range of polymeric vectors for targeted ASO and gene delivery. However, the effectiveness of these therapies is often hindered by challenges achieving therapeutic levels of ASOs at their intracellular site of action and by residual toxicity associated with cationic polymers [263]. The development of synthetic, biocompatible, and biodegradable polymers with controlled and tunable anionic/cationic balance represents a promising step forward in improving ASO delivery. Optimising the charge properties of these polymers would enhance their interaction with ASOs, resulting in increased stability and improved cellular uptake while minimising potential toxicity [264]. Additionally, it could pave the way for site-specific delivery by leveraging tailored electrostatic properties to exploit tissue- or cell-specific targeting mechanisms, addressing both efficacy and safety concerns [265–266].

Building on this foundation, exploring novel nanocarriers composed of functional polyesters, poly(β-amino esters), polycarbonates, or poly(amino acid) derivatives offers an exciting avenue for innovation [267–269]. These materials offer biocompatible and degradable platforms while enabling the design of delivery systems with specific morphologies, such as nanoparticles, micelles, or dendritic structures, which can enhance endosomal escape and controlled release, thereby significantly improving the bioavailability of ASOs [270]. In addition, the incorporation of biopolymers and amino acid-derived functionalities could introduce additional therapeutic benefits, such as dual targeting capabilities or improved compatibility with biological environments [271–272]. In parallel, the increasing recognition of immunogenic responses associated with PEG has driven the search for alternative stealth polymers. Recent findings of anti-PEG antibodies in humans, which trigger immunological reactions, highlight the urgency of developing PEG-free formulations [273–274]. Promising alternatives such as polyzwitterions [275], polyoxazolines [276], and polypeptoids [277] offer a way forward, providing stealth properties while reducing the risk of immunogenicity. These polymers not only exhibit excellent pharmacokinetic profiles but also show potential compatibility with ASO delivery systems, especially in long-term or repeat dosing regimens. Therefore, their incorporation into ASO therapeutics could address significant safety challenges, creating safer and more effective delivery platforms.

Together, advances in polymer chemistry, self-assembly, and nanotechnology are poised to overcome existing barriers in ASO-based therapies. By integrating tailored charge profiles, innovative stimuli-responsive functionalities, and next-generation stealth strategies, researchers can develop more precise, effective, and biocompatible delivery systems. These efforts will play a critical role in optimizing ASO therapeutics for targeted disease treatment and unlocking their full potential in precision medicine.

## Conclusion

In conclusion, this review has highlighted the significant advancements in polymer-assisted delivery of ASOs over the past decade, underscoring the diverse roles that synthetic polymers play in enhancing the delivery, stability, and therapeutic efficacy of ASOs. These polymers have been instrumental in addressing key challenges associated with ASO delivery, including cellular uptake, endosomal escape, and minimising off-target and cytotoxic effects. Studies have shown that tailored polymer systems, whether through pH-responsive mechanisms or nanoparticle encapsulation, have led to improved gene silencing efficiencies and targeted therapeutic effects, as demonstrated by a range of applications in cancer and neurodegenerative disease models. The advancements in ASO delivery through synthetic polymers are paving the way for more precise disease treatments, marking a pivotal shift towards personalised medicine. The ability to fine-tune polymer–ASO interactions for specific cellular environments opens new opportunities for targeted therapies that can be customised to individual patient profiles, thereby increasing treatment efficacy and reducing side effects.

Ongoing research and innovation in this field are crucial for further optimising ASO-based therapeutics. As polymer science continues to evolve, the development of more sophisticated delivery platforms will likely enhance the specificity and safety of ASO treatments, offering transformative potential for treating a wide range of diseases at the molecular level. The continuous exploration of polymer–ASO systems underscores the importance of interdisciplinary research in advancing the future of precision medicine.

## Data Availability

Data sharing is not applicable as no new data was generated or analyzed in this study.
